# Incorporating Lesion-Level Heterogeneity in a Branching-Process Model of Ovarian Cancer Metastasis

**DOI:** 10.1007/s11538-026-01772-7

**Published:** 2026-10-21

**Authors:** Doron Levy

**Affiliations:** Department of Mathematics, University of Maryland, College Park, MD 20742 USA

**Keywords:** Ovarian cancer, Metastasis, Multi-type branching process, Chinese restaurant process, Preferential attachment, Miliary versus bulky phenotype, Tumor heterogeneity, Primary 60J85, 92C50, Secondary 60G42, 60K35, 60K37

## Abstract

Danesh et al. (*J. Theor. Biol.*, 2012) estimated the ovarian cancer screening window at roughly 2.9 years, using a multi-type branching process and the hitting time of an aggregate mass threshold. We extend the model of Danesh et al. to incorporate the lesion-level heterogeneity characteristic of intraperitoneal metastasis, replacing the homogeneous secondary compartment by a vector of distinct lesions whose formation follows a Chinese Restaurant Process with concentration parameter α. We prove three results. Because established metastases divide faster than the peritoneal source compartment, the rate of *de novo* lesion formation is almost surely integrable, so only finitely many lesions ever form and no Yule–Simon power law emerges. Mapping the system to a time-inhomogeneous Hoppe–Polya urn yields a martingale whose almost-sure convergence fixes relative lesion masses at values set by early stochastic events. Kurtz fluid limits give a closed-form trajectory for the largest lesion, whose hitting time of the radiological resolution threshold replaces aggregate mass as the clinical endpoint. A parameter-free criterion follows: the disease is radiologically detectable before it becomes lethal precisely when the largest lesion’s share of the total metastatic mass exceeds the ratio of the imaging resolution limit to the lethal burden. Simulation validates each result and shows the phenotype is governed jointly by α and the peritoneal migration intensity. Calibrating against published surgical series, a fit to the reported prevalence of miliary disease reproduces the observed implant-size dichotomy out of sample and predicts a monotone decline of the screening window with lesion count.

## Introduction and Biological Context

The high mortality rate associated with epithelial ovarian cancer is intrinsically linked to its unique mode of metastasis. Unlike many solid tumors that metastasize primarily via the vasculature, ovarian cancer spreads primarily through direct exfoliation. Cells detach from the primary tumor on the ovary or fallopian tube, enter the fluid of the peritoneal cavity, and eventually implant onto the mesothelial lining of the peritoneum, omentum, and local bowel serosa (Kurman and Shih 2010; Lengyel 2010).

A central challenge in reducing ovarian cancer mortality is establishing a reliable window of opportunity for clinical screening. This window is commonly defined as the time interval between the point a primary tumor becomes detectable via transvaginal ultrasound or CA-125 biomarkers, and the point at which the metastatic burden reaches a lethal threshold (Havrilesky et al. 2008). Mathematical modeling of stochastic population dynamics, particularly continuous-time branching processes, has become a critical tool for estimating this window (Durrett 2015). A landmark study by Danesh et al. (2012) utilized a multi-type branching process to estimate this window at roughly 2.9 years, concluding that screening must occur at least biennially to be effective.

However, existing mathematical models, including the Danesh et al. framework (Danesh et al. 2012), often treat the metastatic burden as a single, homogenous cell population. They ignore the observed lesion-level heterogeneity, where disease presentation ranges from localized bulky masses to widespread, sand-like miliary nodules (Bowtell et al. 2015). Recent advances underscore that capturing this variability is essential, as spatial morphology is deeply intertwined with metabolic reprogramming, drug-tolerant persister cell emergence, and complex tumor microenvironment dynamics (Cunha et al. 2023; Russo et al. 2024).

A terminological clarification is in order at the outset. The extension developed here introduces heterogeneity in the *sizes and number* of distinct metastatic lesions, but it does not track their anatomical positions: there is no metric, no distance-dependent seeding kernel, and no explicit geometry. It is therefore a lesion-level, exchangeable-partition model rather than a spatial model in the usual mathematical or biophysical sense, and we use the term “lesion-level heterogeneity” throughout.

The present paper retains the Danesh et al. three-compartment architecture (primary site, peritoneal fluid, secondary metastatic surface) and the parameter values calibrated in that work, but replaces the homogeneous secondary compartment by a structured ensemble of distinct lesions whose formation is governed by a Chinese Restaurant Process (Aldous 1985; Pitman 2006). Within this extended framework we establish six results that materially affect the Danesh et al. conclusion.

(i) A freezing theorem (Theorem 1, Corollary 1) showing that the asymmetry $\lambda _2 > \lambda _1$, which Danesh et al. themselves identify as biologically necessary, almost surely forces the number of distinct metastatic lesions to remain finite.
(ii) A discrete-index scaling lemma (Lemma 1) converting the supercritical-branching martingale limits into a sample-path bound for the urn-time analysis.
(iii) A martingale-convergence theorem (Theorem 2) showing that the relative mass shares of established lesions stabilize almost surely, with early stochastic events permanently dictating macroscopic structure.
(iv) A fluid-limit derivation (Sect. 6) of an explicit hitting-time formula for the largest individual lesion, which we propose as the appropriate clinical endpoint in place of the Danesh et al. aggregate mass.
(v) A systematic simulation study (Sect. 7) that validates each analytic result quantitatively and establishes that the phenotype is governed jointly by α and the migration intensity.
(vi) A calibration of the model against published surgical series (Sect. 8), fit to lesion counts and validated out of sample against implant sizes.

The extension thereby resolves a degree of freedom that the aggregate-mass formulation integrates out. Write $Y_{\max }$ for the limiting fraction of the total metastatic mass held by the largest single lesion, a quantity made precise in Theorem 2. Two patients with identical aggregate burden *M*(*t*) may have very different $Y_{\max }$, and it is $Y_{\max }$, not the total burden, that determines whether a lesion crosses the radiological resolution limit before the aggregate burden becomes lethal.

The paper is organized as follows. Section 2 summarizes the baseline Danesh et al. branching process model and collects its parameter values in Table 1. Section 3 formally defines the CRP extension. Section 3.4 gives a complete specification of the stochastic process in terms of its transition rates. Section 4 derives the mean-field size-distribution equations, proves the breakdown of the Yule–Simon power-law regime, and establishes the freezing theorem. Section 5 presents an exact stochastic formulation using infinite-color time-non-homogeneous Polya urns and martingales. Section 6 applies fluid limits to calculate deterministic tumor trajectories and hitting times, conditioning on the freezing event so that the standard density-dependent theory applies. Sect. 7 reports a systematic simulation study validating each analytic result. Section 8 calibrates the model against published surgical series and states the resulting falsifiable predictions. Sect. 9 discusses the biological and clinical implications of the framework, and Sect. 10 concludes with a discussion of future modeling directions.

**Table 1:** Model parameters, interpretation and provenance. Rates are in $\hbox {month}^{-1}$. Thresholds and burdens are absolute cell counts

Symbol	Value	Interpretation	Source
$\lambda _0$	0.173	Primary tumor net growth rate	4-month doubling Danesh et al. (2012)
$\lambda _1$	0.173	Peritoneal net growth rate	$\lambda _1 \approx \lambda _0$ Danesh et al. (2012)
$\lambda _2$	0.277	Metastatic net growth rate	2.5-month doubling Danesh et al. (2012)
$\lambda _1/\lambda _2$	0.625	Growth asymmetry	Derived
$u_1$	Structural	Exfoliation rate per primary cell	Danesh et al. (2012)
$u_2$	Structural	Attachment rate per peritoneal cell	Danesh et al. (2012)
$u_0 = u_2 Z_1(0)$	0.1–100	Migration intensity at t=0	Free parameter, swept
α	0.5–1000	CRP concentration (mesothelial receptivity)	Free parameter, swept
$M_0$	$10^5$–$10^7$	Primary detection threshold	Danesh et al. (2012)
$C_{\text {detect}}$	$\sim 10^8$	Imaging resolution, ∼1 cm nodule	Sect. 6
$M_{\text {lethal}}$	$10^9$	Lethal aggregate burden, $\sim 1\,\hbox {cm}^3$	Danesh et al. (2012)
*R*	10	Detection-to-lethality ratio	Derived; see Sect. 7.6

## The Danesh et al. Model

The work of Danesh et al. (2012) represents a milestone in the application of multi-type continuous-time Markov branching processes (CTMBP) to gynecological oncology (Kimmel and Axelrod 2002). The core objective of their study was to calculate a biologically realistic window of opportunity for ovarian cancer screening by translating clinical tumor doubling times into precise stochastic parameters, expanding upon traditional multi-event theories of carcinogenesis (Moolgavkar and Venzon 1979).

The Danesh et al. model operates on a three-compartment architecture, reflecting the unique physiological journey of ovarian cancer metastasis: the primary site (Type 0), the peritoneal fluid (Type 1), and the secondary metastatic surfaces (Type 2).

### Primary Tumor Dynamics

The primary tumor, located in the fallopian tube or ovary, is modeled as a simple birth-death branching process. Let $Z_0(t)$ denote the number of Type 0 cells at time *t*. Each cell divides at rate $a_0$ and dies at rate $b_0$. The net exponential growth rate is thus defined as $\lambda _0 = a_0 - b_0$. Assuming the tumor initiates from a single malignant cell at time t=0 (i.e., $Z_0(0) = 1$), standard branching process theory dictates that conditioned on non-extinction, the population size grows deterministically in the large-time limit:

1 $$\begin{aligned} Z_0(t) \sim W_0 e^{\lambda _0 t}, \end{aligned}$$

where $W_0$ is a strictly positive random variable representing the early stochastic fluctuations of the initiating cell lineage (Nowak 2006). Based on clinical literature and stem cell division kinetics, the authors establish a primary tumor doubling time of approximately 4 months, which algebraically yields $\lambda _0 = (\ln 2) / 4 \approx 0.173 \text { months}^{-1}$.

### Migration and the Poisson Point Process Approximation

As the primary tumor grows, cells detach and enter the peritoneal cavity. Danesh et al. model this exfoliation as a mutation-like event occurring at a rate $u_1$ per primary cell, drawing on foundational multi-type clonal expansion mechanics (Bozic et al. 2010; Haeno et al. 2007). Therefore, the total rate at which Type 1 (peritoneal) cells are generated at time *t* is $u_1 Z_0(t)$. Because $Z_0(t)$ grows exponentially, the arrival of the first peritoneal cell occurs at a random time $\tau _1$. Using a non-homogeneous Poisson point process approximation, the cumulative rate function is integrated to evaluate the arrival times. The number of Type 1 cells, $Z_1(t)$, then initiates its own birth-death process with division rate $a_1$ and death rate $b_1$. Given that peritoneal cells are floating in ascites fluid, they do not establish vascular networks, leading the authors to assume their net growth rate is no greater than the primary tumor, typically setting $\lambda _1 = a_1 - b_1 \approx \lambda _0$.

The transition from the peritoneal fluid (Type 1) to the solid secondary metastatic sites (Type 2) is the critical bottleneck in the disease. A peritoneal cell successfully attaches to a mesothelial surface (such as the omentum) at a rate $u_2$. The total rate of generating new metastatic cells is thus $u_2 Z_1(t)$.

Once attached, these Type 2 cells exhibit highly aggressive behavior, escaping the constraints of suspension-induced cell death (Iwasa et al. 2006). Angiogenesis and a favorable microenvironment allow them to divide at an accelerated rate $a_2$, with a death rate $b_2$. Danesh et al. parameterize the net metastatic growth rate $\lambda _2 = a_2 - b_2$ based on clinical observations of secondary tumor doubling times of 2.5 months. This yields $\lambda _2 = (\ln 2) / 2.5 \approx 0.277 \text { months}^{-1}$.

### Calculating the Window of Opportunity

A profound mathematical consequence of this parameterization is the strict inequality $\lambda _2 > \lambda _1 \approx \lambda _0$. Because the secondary sites grow exponentially faster than the source sites, the total metastatic mass $Z_2(t)$ rapidly outpaces both the primary tumor and the peritoneal compartment. The deterministic backbone of the total metastatic mass satisfies the limiting differential equation:

2 $$\begin{aligned} \frac{dZ_2(t)}{dt} = \lambda _2 Z_2(t) + u_2 Z_1(t). \end{aligned}$$

The clinical window of opportunity is defined as the time difference $T_2 - T_0$. Here, $T_0$ is the time at which the primary tumor reaches a radiologically detectable volume (defined as $M_0 = 10^5$ to $10^7$ cells, roughly the resolution of transvaginal ultrasound). $T_2$ is the time at which the aggregate metastatic burden reaches a lethal threshold, defined as $M_2 = 10^9$ cells (corresponding to approximately $1\,\text {cm}^3$ of tumor tissue under the commonly quoted conversion $1\,\text {cm}^3 \approx 10^9$ cells).

By evaluating the hitting times of these stochastic limits, Danesh et al. derived the expected window of opportunity. They calculated that it takes, on average, 2.9 years from the moment the primary tumor is $10^5$ cells until the metastatic mass reaches $10^9$ cells. Under the assumptions of their aggregate-mass model, this calculation provides a quantitative basis for the clinical guideline that ovarian cancer screening must occur at least once every two years to be effective: annual screening is preferred, while triennial screening is predicted to be largely ineffective under the model, as the entire window of opportunity could open and close between checkups.

For reference, Table 1 collects every parameter used in this paper, together with its interpretation and provenance. We draw attention to one point that will matter in Sect. 7: the exfoliation and attachment rates $u_1, u_2$ are structural in the Danesh et al. formulation but are not pinned to a unique numerical value there. In our analysis they enter only through the migration intensity $u(t) = u_2 Z_1(t)$ at the start of the metastatic phase, which we denote $u_0:= u_2 Z_1(0)$ and treat as a free parameter.

## Lesion-Level Heterogeneity via the Chinese Restaurant Process

While the Danesh et al. framework provides an essential temporal timeline for disease progression, its fundamental limitation lies in its topological uniformity. The model assumes that all cells transitioning from the peritoneum into the Type 2 state, pool into a single, aggregate mass $Z_2(t)$. It effectively models metastasis as a single continually growing mass. We replace this transition from the peritoneum into the Type 2 state with a Chinese Restaurant Process, as described below.

### Defining the State Space

Let the lesion-level configuration of the metastatic burden at time *t* be represented by a state vector of fluctuating length:

3 $$\begin{aligned} \textbf{X}(t) = (X_1(t), X_2(t), \dots , X_{L(t)}(t)), \end{aligned}$$

where $X_i(t)$ denotes the number of cancer cells comprising the *i*-th distinct metastatic lesion, and *L*(*t*) represents the total number of distinct lesions established at time *t*. The aggregate metastatic mass evaluated by Danesh et al. is recovered by the sum $M(t) = \sum _{i=1}^{L(t)} X_i(t)$.

Upon surgical exploration of the abdominal cavity, the topological distribution of advanced-stage disease rarely presents as a singular, coalesced mass. Rather, macroscopic evaluation at laparotomy reveals highly heterogeneous, distinct lesions scattered across distinct anatomical regions (Makar et al. 2016). Some patients present with a *bulky* phenotype, characterized by a few massive, heavily vascularized tumors typically localized to the greater omentum. In stark contrast, other patients exhibit a *miliary* presentation, defined by thousands of diffuse, sand-like nodules blanketing the peritoneal lining, diaphragmatic undersurface, and bowel serosa (Schnaiter et al. 2024).

### Biological Justification for the CRP Mechanism

We model the transition of a cell from the peritoneal fluid to the metastatic state via the rules of the Chinese Restaurant Process (CRP). The CRP is a discrete-time stochastic process that generates a distribution over partitions of a set, typically utilized in Bayesian nonparametrics (Blackwell and MacQueen 1973; Blei et al. 2010). In our continuous-time biological adaptation, arriving migrating cells act as “customers,” and the distinct spatial lesions act as “tables.”

When a cell successfully navigates the peritoneal fluid and prepares to implant on the mesothelial surface, its attachment location is not chosen uniformly at random. We define two competing topological behaviors, modulated by a tissue-specific concentration parameter α>0:

1. *Preferential attachment (Joining an existing lesion):* Tumors are not passive masses. They actively modulate their microenvironment. Established lesions upregulate adhesion molecules (such as integrins and cadherins), secrete inflammatory cytokines, and develop complex angiogenic vascular networks that provide structural and signaling support for incoming cells (Geho et al. 2005). These biological factors act as a localized sink, actively attracting and capturing circulating cells. To model this, the probability that an incoming cell attaches to an existing lesion *i* is strictly proportional to the lesion’s current cellular mass:
  4 $$\begin{aligned} P(\text {Attach to lesion } i) = \frac{X_i(t)}{M(t) + \alpha }. \end{aligned}$$
  This implements a rigorous “rich-get-richer” dynamic. A tumor that becomes large early in the disease progression will attract subsequent migrating cells.
2. *De Novo seeding (Starting a new lesion):* Alternatively, a cell may implant on a previously unoccupied section of the mesothelium, thereby seeding a brand-new distinct lesion, which increments the state vector length L(t)→L(t)+1. The probability of this occurring is governed by the concentration parameter:
  5 $$\begin{aligned} P(\text {Seed new lesion}) = \frac{\alpha }{M(t) + \alpha }. \end{aligned}$$

### The Concentration Parameter α

The parameter α is of paramount biological significance in this framework. It quantifies the innate permissiveness of the patient’s healthy peritoneal tissue to *de novo* implantation versus the chemotactic draw of existing tumors. If α is infinitesimally small ($\alpha \rightarrow 0$), the healthy mesothelium is highly hostile to initial implantation, and arriving cells are forced to aggregate onto established tumor masses. This mathematically forces the system into a bulky spread phenotype. Conversely, if α is large ($\alpha \rightarrow \infty$), the entire peritoneal cavity is highly receptive to seeding, overwhelming the preferential attachment mechanic and resulting in thousands of independent micro-lesions (miliary spread). By tuning this single parameter, our model encompasses the entire topological spectrum of the disease.

**Fig. 1:**
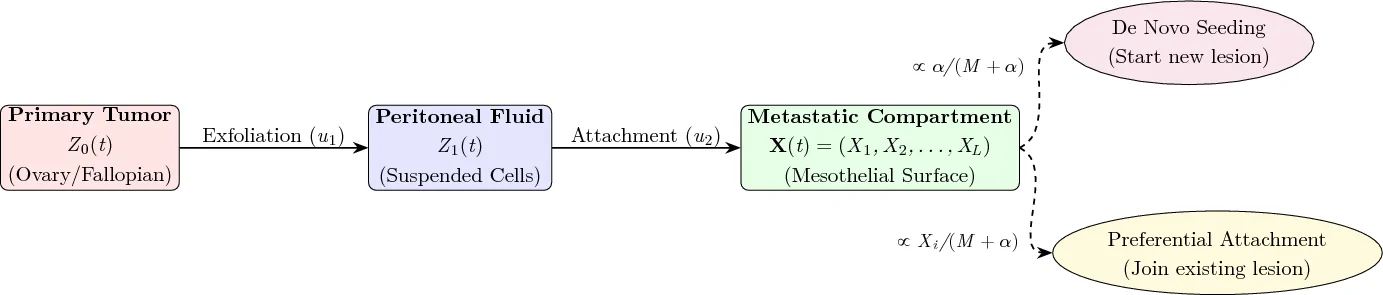
System schematic detailing cellular navigation through the three-compartment model. Cells exfoliate from the primary architecture into suspension ($Z_1$), subsequently implanting onto the secondary mesothelial lining. The topological arrangement of these secondary masses is governed explicitly by the competing probabilities of preferential attachment and de novo seeding modulated by α

A visual overview of this structural layout, which integrates the continuous-time multi-type branching equations with the rules of the Chinese Restaurant Process, is presented in Fig. 1.

As shown in Fig. 1, incoming cells traversing the peritoneal fluid are captured by the secondary compartment via two competing kinetic pathways. To replace the monolithic compartment $Z_2(t)$ with a vector-valued state, we govern these dynamics by the principles of preferential attachment.

### Specification of the Stochastic Process

The state at time *t* is the triple

6 $$\begin{aligned} S(t) \;=\; \bigl (Z_0(t),\, Z_1(t),\, \textbf{X}(t)\bigr ), \qquad \textbf{X}(t) = (X_1(t),\dots ,X_{L(t)}(t)) \in \bigcup _{\ell \ge 0} \mathbb {N}^{\ell }, \end{aligned}$$

where the last component has fluctuating length *L*(*t*) and $M(t) = \sum _{i=1}^{L(t)} X_i(t)$. The process is a continuous-time Markov jump process on this state space with the following six transitions:

**Table:** 

	Event	Rate	Effect on the state
(T1)	Primary division	$a_0 Z_0$	$Z_0 \rightarrow Z_0 + 1$
(T2)	Primary death	$b_0 Z_0$	$Z_0 \rightarrow Z_0 - 1$
(T3)	Exfoliation	$u_1 Z_0$	$Z_1 \rightarrow Z_1 + 1$
(T4)	Peritoneal birth/death	$a_1 Z_1$ / $b_1 Z_1$	$Z_1 \rightarrow Z_1 \pm 1$
(T5)	Metastatic division	$\lambda _2 X_i$	$X_i \rightarrow X_i + 1$
(T6a)	Attachment to lesion *i*	$u_2 Z_1 \cdot \dfrac{X_i}{M+\alpha }$	$X_i \rightarrow X_i + 1$
(T6b)	*De novo* seeding	$u_2 Z_1 \cdot \dfrac{\alpha }{M+\alpha }$	L→L+1, $X_{L+1} = 1$

Three features of this specification deserve emphasis, because they are the source of every subsequent result.

First, transitions (T5) and (T6) are *distinct channels with different consequences*. Endogenous division (T5) selects a cell uniformly from the *M* cells already attached, so lesion *i* is chosen with probability $X_i/M$. A dividing cell remains within its own lesion and can never create a new one. Only migration (T6) is routed through the CRP rule, and only (T6b) can increase *L*. Collapsing these two channels (T5 and T6) into a single CRP draw, that is, treating every cell addition as a restaurant customer, yields a *different* process, namely a plain Hoppe urn, in which *L* grows without bound like $\alpha \log M$. The freezing phenomenon established in Sect. 4 depends on keeping them distinct.

Second, the total propensity for the metastatic compartment to grow is

7 $$\begin{aligned} \Omega (t) \;=\; \underbrace{\lambda _2 M(t)}_{\text {endogenous}} \;+\; \underbrace{\mathrm{u(t)}}_{\text {exogenous}}\!\!\!\!\!, \qquad u(t) := u_2 Z_1(t), \end{aligned}$$

and the competition between these two terms is the mechanism of the entire paper. Since *M*(*t*) grows at rate $\lambda _2$ while *u*(*t*) grows at the strictly slower rate $\lambda _1$, the exogenous share u(t)/Ω(t) decays to zero, and with it the only channel capable of creating lesions.

Third, α enters *only* through (T6a)–(T6b), and only as the relative weight of seeding against attachment within the migration channel. It does not affect the growth of any established lesion, nor the total mass trajectory *M*(*t*), which is why the aggregate-mass predictions of Danesh et al. are recovered exactly by summing over *i* regardless of α.

### Scope of the Construction: What is and is Not Modeled

Because the CRP generates an exchangeable partition, the model is invariant under relabeling of lesions. No lesion has a position, and no pair of lesions has a distance. What the construction does capture is the *size distribution* induced by locally reinforced implantation: a cell arriving in a cavity that already contains large, well-vascularized, adhesion-competent implants is more likely to be captured by one of them than to found a new colony on bare mesothelium, and the capture probability scales with implant mass. In that sense the CRP is a mean-field proxy for spatial structure. It reproduces the consequences of spatially localized capture for the lesion-size hierarchy without tracking where the lesions are.

What is lost is correspondingly explicit. The model cannot represent distance-dependent seeding, in which a cell shed near the omentum is more likely to implant there than on the diaphragm. It cannot represent anatomical site specificity, and in particular cannot distinguish the omentum, which is highly receptive, from the small bowel serosa, which is not. Nor can it represent gravitational or flow-driven pooling of ascites, which concentrates implantation in the pouch of Douglas, the right paracolic gutter and the right subphrenic space (van Baal et al. 2018). A single tissue-uniform α averages over all of this. Extensions with a site-dependent $\alpha (\textbf{x})$ or an explicit seeding kernel are the natural next step and are discussed in Sect. 10.

We are also explicit that the CRP is adopted as a modeling ansatz rather than derived from implantation biology. Preferential attachment is qualitatively motivated by the adhesion and microenvironmental arguments given in Sect. 3, since established implants upregulate adhesion molecules, secrete cytokines and develop vasculature, and therefore capture arriving cells more efficiently than bare mesothelium. However, we are not aware of direct measurements establishing that the capture rate is *linearly* proportional to implant mass, which is the specific functional form the CRP assumes. Linearity is the simplest form consistent with the qualitative biology. A sublinear capture rule of the form $X_i^{\theta }$ with θ<1 would be equally consistent with the qualitative evidence and would weaken the rich-get-richer effect. We regard discriminating between these alternatives as an empirical question that the framework poses but does not settle.

## Mean-Field Analysis and Power-Law Breakdown

Throughout the rigorous statements that follow, we work on the standard probability space $(\Omega , \mathcal {F}, P)$ on which the branching processes $Z_0(t)$, $Z_1(t)$, and *M*(*t*) are jointly defined, with $\mathcal {F}_t$ denoting the natural filtration generated by the process history up to time *t*. We let $\Omega ^* \subseteq \Omega$ denote the *non-extinction event*: the set of sample paths along which all three branching compartments grow to infinity, equivalently the set on which $M(t) \rightarrow \infty$ as $t \rightarrow \infty$. Since each compartment is a supercritical birth–death process with division rate strictly exceeding death rate ($a_i > b_i$, i=0,1,2), standard branching-process theory gives $P(\Omega ^*) = \prod _{i} (1 - b_i/a_i) > 0$, and on the complement $\Omega \setminus \Omega ^*$ the disease ceases stochastically before becoming clinically detectable. All almost-sure statements in what follows are understood to hold conditionally on $\Omega ^*$, which is the only event of clinical relevance: patients whose nascent tumor goes extinct before becoming a clinical entity are excluded.

To evaluate the asymptotic topological structure generated by this CRP extension, we construct a deterministic mean-field approximation. This allows us to track the expected distribution of lesion sizes over time.

Let $N_k(t)$ represent the expected number of distinct metastatic lesions containing exactly *k* cells at time *t*. The dynamics of the system are governed by two independent forces: endogenous exponential cellular division occurring strictly within the secondary sites at rate $\lambda _2$, and exogenous cellular migration arriving from the peritoneum at a time-dependent rate $u(t) = u_2 Z_1(t)$.

### Deriving the Mean-Field Size-Distribution Equations

We define the transition rates for individual lesions. For a specific lesion that currently possesses *k* cells, the rate $r_k(t)$ at which it grows to size k+1 is the sum of its internal division rate and its external capture rate (governed by the CRP preferential attachment rule):

8 $$\begin{aligned} r_k(t) = \lambda _2 k + u(t) \frac{k}{M(t) + \alpha }. \end{aligned}$$

Similarly, the rate $r_{\text {new}}(t)$ at which entirely new, single-cell lesions are seeded onto the mesothelium is determined by the total influx rate multiplied by the CRP *de novo* probability:

9 $$\begin{aligned} r_{\text {new}}(t) = u(t) \frac{\alpha }{M(t) + \alpha }. \end{aligned}$$

By balancing the inflow and outflow of lesions of specific sizes, we construct an infinite, coupled system of ordinary differential equations for the expected counts: We note that these are *not* master equations in the standard sense. A master equation governs the evolution of the full probability distribution over states of the stochastic process, whereas the system below governs only the first moments $N_k(t) = \mathbb {E}[\#\{i: X_i(t) = k\}]$, and is obtained by replacing the products of random variables appearing in the exact moment hierarchy with products of their expectations. It is therefore a mean-field (moment-closure) approximation. The exact stochastic treatment, which does not rely on this closure, is given in Sect. 5.

10 $$\begin{aligned} \frac{dN_1(t)}{dt}&= r_{\text {new}}(t) - N_1(t) r_1(t), \end{aligned}$$

11 $$\begin{aligned} \frac{dN_k(t)}{dt}&= N_{k-1}(t) r_{k-1}(t) - N_k(t) r_k(t), \quad \text {for } k \ge 2. \end{aligned}$$

### The Classical Yule–Simon Limit

To understand the novelty of the ovarian cancer application, it is instructive to first consider what these equations predict under the classical network-theoretic scaling that produces scale-free distributions (Barabási and Albert 1999; Simon 1955; Yule 1925). The classical Yule–Simon setting assumes that the rate of new arrivals is proportional to the current size of the system. In our notation, this corresponds to taking u(t)=cM(t) for some constant c>0, so that the migration influx co-scales with the current metastatic mass.

Under this hypothesis, in the large-mass limit $M(t) \gg \alpha$ the CRP denominator simplifies ($M(t) + \alpha \approx M(t)$) and the per-lesion transition rates linearize:

12 $$\begin{aligned} r_k(t)&\approx (\lambda _2 + c)\,k, \end{aligned}$$

13 $$\begin{aligned} r_{\text {new}}(t)&\approx c\alpha . \end{aligned}$$

The rate at which individual cells are added to the total mass is $(\lambda _2+c)M(t)$, while new lesions are created at rate cα. Treating the system per cell-addition event, the probability that an added cell starts a new lesion is

14 $$\begin{aligned} p(t) = \frac{c\alpha }{(\lambda _2+c)M(t) + c\alpha } \;\xrightarrow {M\rightarrow \infty }\; 0. \end{aligned}$$

Because p→0 rather than approaching a positive constant, a strict Yule–Simon stationary distribution does not exist in this regime. However, if one nonetheless seeks a self-similar profile $N_k(t) = L(t)\,p_k$ in the regime where *L*(*t*) grows slowly relative to *M*(*t*), the balance equations

15 $$\begin{aligned} \frac{dL}{dt}\,p_1&= c\alpha - L(\lambda _2+c)\,p_1, \end{aligned}$$

16 $$\begin{aligned} \frac{dL}{dt}\,p_k&= L(\lambda _2+c)\bigl [(k-1)p_{k-1} - k\,p_k\bigr ], \quad (k\ge 2), \end{aligned}$$

combined with dL/dt=cα, yield a recurrence whose asymptotic solution is a Pareto-type tail

17 $$\begin{aligned} p_k \sim k^{-\gamma }, \qquad \gamma = 1 + \frac{\lambda _2 + c}{c}, \end{aligned}$$

valid in a quasi-stationary sense (Simon 1955). This serves as the natural reference point for assessing whether ovarian metastasis admits a heavy-tailed lesion-size hierarchy. As we now show, the actual biological scaling fails the key hypothesis u(t)=cM(t) that underwrites this construction, and the resulting departure from scale-free behavior is evident.

### The Durrett Asymmetry and Theorem 1

The biological parameters derived in the Danesh et al. framework force a breakdown of this classical power law. In ovarian cancer, the influx rate *u*(*t*) does not scale with the metastatic mass *M*(*t*). The influx is driven entirely by the peritoneal population $Z_1(t)$.

As established in Sect. 2, $Z_1(t)$ grows at a rate dictated by the primary tumor ($\lambda _1 \approx 0.173$), meaning $u(t) \sim \mathcal {O}(e^{\lambda _1 t})$. Simultaneously, the total metastatic mass *M*(*t*) undergoes rapid, aggressive internal division at rate $\lambda _2 \approx 0.277$, meaning $M(t) \sim \mathcal {O}(e^{\lambda _2 t})$.

Because clinical data suggests that metastatic cells divide faster and are more proliferative than primary cells ($\lambda _2 > \lambda _1$), as $t \rightarrow \infty$, the ratio of the migration rate to the internal mass collapses to zero:

18 $$\begin{aligned} \lim _{t \rightarrow \infty } \frac{u(t)}{M(t)} = \lim _{t \rightarrow \infty } \frac{\mathcal {O}(e^{\lambda _1 t})}{\mathcal {O}(e^{\lambda _2 t})} = \lim _{t \rightarrow \infty } \mathcal {O}(e^{(\lambda _1 - \lambda _2)t}) = 0. \end{aligned}$$

#### Theorem 1

(Breakdown of the Power Law and Freezing of Topology). In a multi-type branching model of metastasis where the secondary division rate exceeds the primary proliferation rate ($\lambda _2 > \lambda _1$), the cumulative rate of new lesion formation is integrable: $\int _0^\infty r_{\text {new}}(t)\,dt < \infty$. Consequently, the expected total number of distinct metastatic lesions $\mathbb {E}[L(\infty )]$ is finite, and the assumptions under which a Yule–Simon power-law lesion-size distribution emerges are violated.

#### Proof

We first record the pointwise limit of the formation rate. Substituting the asymptotic ratio derived in Equation (18),

19 $$\begin{aligned} \lim _{t \rightarrow \infty } r_{\text {new}}(t) = \lim _{t \rightarrow \infty } u(t) \frac{\alpha }{M(t) + \alpha } = \alpha \lim _{t \rightarrow \infty } \frac{u(t)}{M(t)} = 0. \end{aligned}$$

We will now establish almost sure of the *rate* of decay.

Both the peritoneal influx $u(t) = u_2 Z_1(t)$ and the metastatic mass *M*(*t*) are driven by supercritical branching processes with growth rates $\lambda _1$ and $\lambda _2$ respectively. By the Kesten–Stigum / martingale limit theorem for supercritical branching processes (Athreya and Ney 1972), on the event of non-extinction $\Omega ^*$ (the only event of clinical relevance) there exist strictly positive, almost surely finite random variables $W_1, W_2 > 0$ such that

20 $$\begin{aligned} e^{-\lambda _1 t} Z_1(t) \xrightarrow {\text {a.s.}} W_1, \qquad e^{-\lambda _2 t} M(t) \xrightarrow {\text {a.s.}} W_2 \qquad (t \rightarrow \infty ). \end{aligned}$$

Fix a realization in $\Omega ^*$. By (20) there is an almost surely finite random time $T_0$ and random constants $0< c_2 \le C_1 < \infty$ such that $u(t) \le C_1 e^{\lambda _1 t}$ and $M(t)+\lambda \ge M(t)+\lambda \ge M(t) \ge c_2e^{\lambda _2t}$ for all $t \ge T_0$. Hence, for all $t \ge T_0$,

21 $$\begin{aligned} r_{\text {new}}(t) = \alpha \,\frac{u(t)}{M(t) + \alpha } \;\le \; \frac{\alpha C_1}{c_2}\, e^{(\lambda _1 - \lambda _2)t} \;=:\; K\, e^{(\lambda _1 - \lambda _2)t}, \end{aligned}$$

where $K = \alpha C_1 / c_2$ is a random but almost surely finite constant. Since $\lambda _1 - \lambda _2 < 0$, this dominating exponential is integrable on $[T_0, \infty )$, while $r_{\text {new}}$ is bounded and continuous on the finite interval $[0, T_0]$. Therefore, almost surely on $\Omega ^*$,

22 $$\begin{aligned} \int _0^\infty r_{\text {new}}(t)\,dt \;\le \; \int _0^{T_0} r_{\text {new}}(t)\,dt \;+\; \frac{K}{\lambda _2 - \lambda _1}\, e^{(\lambda _1 - \lambda _2)T_0} \;<\; \infty . \end{aligned}$$

The integral is finite for every realization in $\Omega ^*$. The value of the bound is random through $T_0$ and *K*, but its finiteness is not.

In the inhomogeneous-Poisson description in which new lesions are seeded at the (history-dependent) rate $r_{\text {new}}(t)$, the conditional expected number of lesions formed over the patient’s lifetime equals $\int _0^\infty r_{\text {new}}(t)\,dt$, which we have just shown is almost surely finite on non-extinction. Consequently $\mathbb {E}[L(\infty )\mid \Omega ^*] < \infty$. The classical Yule–Simon hypothesis u(t)∝M(t) is therefore inconsistent with the biological scaling $u(t) \sim e^{\lambda _1 t}$, $M(t) \sim e^{\lambda _2 t}$ with $\lambda _2 > \lambda _1$: the exponential expansion of established lesions functionally monopolizes the CRP attachment denominator and deprives the healthy mesothelium of new migrating cells. The strengthening to almost-sure finiteness of L(∞) itself (rather than of its conditional expectation) is established separately in Corollary 1 via Borel–Cantelli. □

The almost-sure growth control established in the proof above has a discrete-index counterpart that we isolate here, since both the Polya-urn martingale analysis (Theorem 2) and the Borel–Cantelli finiteness argument (Corollary 1) of Sect. 5 index the system by cumulative cell count *n* rather than by time *t*, and both require the migration term $u(t_n)$ as a function of *n*.

#### Lemma 1

(Almost-sure discrete-index scaling). Define the step index by $M(t_n) = n$, so that $t_n$ is the (random) time at which the metastatic compartment first contains *n* cells. On the non-extinction event $\Omega ^*$, and conditionally on $\lambda _2> \lambda _1 > 0$, there exist almost surely finite, strictly positive random constants *c*, *C* and a finite random index $n_0$ such that

23 $$\begin{aligned} c\, n^{\lambda _1/\lambda _2} \;\le \; u(t_n) \;\le \; C\, n^{\lambda _1/\lambda _2} \qquad \text {for all } n \ge n_0, \quad \text {a.s. on } \Omega ^*. \end{aligned}$$

#### Proof

By the martingale limits (20), on $\Omega ^*$ we have $M(t) = W_2 e^{\lambda _2 t}(1 + o(1))$ and $u(t) = u_2 Z_1(t) = u_2 W_1 e^{\lambda _1 t}(1 + o(1))$ almost surely, with $W_1, W_2 > 0$. Imposing the defining relation $M(t_n) = n$ and inverting gives, almost surely as $n \rightarrow \infty$,

24 $$\begin{aligned} \log n&= \log W_2 + \lambda _2 t_n + o(1) \implies t_n = \frac{1}{\lambda _2}\log n - \frac{1}{\lambda _2}\log W_2 + o(1) \nonumber \\ &= \frac{1}{\lambda _2}\log n + O_{\text {a.s.}}(1). \end{aligned}$$

Since $\log W_2$ is a.s. finite on $\Omega ^*$, the term $- \frac{1}{\lambda _2}\log W_2 + o(1)$ is $O_{\text {a.s.}}(1)$. Substituting into the expression for *u*,

25 $$\begin{aligned} u(t_n)&= u_2 W_1 \exp \!\left( \lambda _1 t_n\right) (1+o(1)) \nonumber \\&= u_2 W_1 \exp \!\left( \frac{\lambda _1}{\lambda _2}\log n + O_{\text {a.s.}}(1)\right) (1+o(1)) \nonumber \\&= u_2 W_1 W_2^{-\lambda _1/\lambda _2}\, n^{\lambda _1/\lambda _2}\,(1+o(1)) \;\asymp \; n^{\lambda _1/\lambda _2}, \end{aligned}$$

where the implied constants in the ≍ relation are given by the random prefactor $u_2 W_1 W_2^{-\lambda _1/\lambda _2}$, a function of $W_1$ and $W_2$ that is almost surely strictly positive and almost surely finite on $\Omega ^*$ (since $W_1, W_2 > 0$ a.s. there). This yields (23). □

With Lemma 1 in hand, every appearance of the heuristic scaling “$u(t_n) \sim n^{\lambda _1/\lambda _2}$” in the sequel is to be read as the almost-sure two-sided bound (23): the implied constants are random, but their finiteness and positivity, which is all the convergence and summability arguments use, hold for every sample path on the clinically relevant event $\Omega ^*$. We make this substitution silently in Theorem 2 and Corollary 1 to avoid repeating the conditioning argument. See also caveat (iii) of Sect. 5.4.

## Analytic Formulation via Polya Urns and Martingales

While mean-field approximations reveal average macroscopic trajectories, they obscure the underlying stochastic fluctuations and sample-path dependencies critical to early-stage oncogenesis. The clinical reality of cancer is heavily dependent on early stochastic events that cascade into downstream consequences. To capture these dynamics we map our process onto an infinite-color, *time-non-homogeneous* Hoppe–Polya urn scheme (Hoppe 1984). While the Hoppe urn / Ewens / CRP equivalence is itself classical, the genuine novelty of our construction is the time-inhomogeneous migration mechanism driven by the separate, exponentially-growing peritoneal compartment $Z_1(t)$. It is precisely this coupling between two distinct growth rates ($\lambda _1$ for arrivals, $\lambda _2$ for in-place division) that produces the freezing of de novo lesion formation established below, and that distinguishes the present model from standard reinforced-urn or preferential-attachment systems.

### Urn Scheme Mapping

Let individual metastatic cells represent “balls” contained within an urn. Distinct metastatic lesions represent unique “colors.” The concentration parameter α is represented by a special mutable black ball of mass α permanently residing in the urn. The black ball allows a single mass-weighted urn draw to express both CRP mechanisms simultaneously. At each step a ball is drawn with probability proportional to its mass (the total urn mass at step *n* is n+α). Drawing one of the $X_i(n)$ colored balls of color *i* occurs with probability $X_i(n)/(n+\alpha )$ and triggers preferential attachment (a new ball of the same color is added, reinforcing lesion *i*). Drawing the black ball occurs with probability $\alpha /(n+\alpha )$ and triggers *de novo* seeding (a new ball of a previously unused color is added, creating lesion L(n)+1). The two CRP outcomes are thus collapsed into a single urn operation rather than specified as two separate exogenous processes, which preserves exchangeability and grants access to the standard machinery of Hoppe–Pólya urns: the Ewens partition structure under stationary migration (Ewens 1972; Hoppe 1984), and the reinforced-urn martingale theory underlying Theorem 2.

A note on terminology: we describe the black ball as “mutable” because, although its intrinsic mass α is constant, its *relative* weight in the urn evolves with the system. Early in the disease, when *M*(*n*) is comparable to α, the black ball comprises a substantial fraction of total urn mass and *de novo* seeding competes effectively with preferential attachment. As $M(n) \rightarrow \infty$, the black ball’s mass fraction $\alpha /(n+\alpha ) \rightarrow 0$ and innovation events become rare. This dilution, in combination with the migration rate $u(t_n)$ decaying as established in Sect. 4, is the probabilistic mechanism behind the freezing of *de novo* lesion creation: the black ball is not removed from the urn, but the growing colored mass effectively starves it of opportunities to be drawn.

Instead of tracking continuous time *t*, we map the system to a discrete step sequence $n \in \mathbb {N}$, indexing the total number of metastatic cells, such that $M(t_n) = n$. At each state transition n→n+1, a single cell is added to the metastatic compartment. This addition can occur via two competing mechanisms: an internal cellular division (endogenous), or an external migration event from the peritoneal cavity (exogenous).

The total propensity for system expansion at step *n* is given by $\Omega (n) = \lambda _2 n + u(t_n)$. We decompose the exact probability $\Pi _i(n)$ that the *n*-th cell joins the *i*-th existing lesion (i.e., is painted with color *i*) as a mixture of its branching propensity and its CRP migration capture propensity:

26 $$\begin{aligned} \Pi _i(n)&= \frac{\lambda _2 X_i(n)}{\lambda _2 n + u(t_n)} + \left( \frac{u(t_n)}{\lambda _2 n + u(t_n)} \right) \left( \frac{X_i(n)}{n + \alpha } \right) \nonumber \\&= \frac{X_i(n)}{\lambda _2 n + u(t_n)} \left[ \lambda _2 + \frac{u(t_n)}{n + \alpha } \right] . \end{aligned}$$

Correspondingly, the probability $\Pi _{\text {new}}(n)$ of drawing the black ball and seeding a brand-new color (a new lesion) is strictly derived from the exogenous migration term, forming a variant of Ewens sampling partitions (Ewens 1972):

27 $$\begin{aligned} \Pi _{\text {new}}(n) = \left( \frac{u(t_n)}{\lambda _2 n + u(t_n)} \right) \left( \frac{\alpha }{n + \alpha } \right) = \frac{\alpha u(t_n)}{(n + \alpha )(\lambda _2 n + u(t_n))}. \end{aligned}$$

### Martingale Construction and Almost Sure Convergence

To verify the structural emergence of dominant tumor sinks (which we define as established lesions that disproportionately capture and accumulate new migrating cells due to their mass advantage), we define the relative mass fraction of the *i*-th lesion at step *n* as $Y_i(n) = X_i(n)/n$. We seek to prove that early stochastic advantages are permanently locked into the system architecture.

#### Theorem 2

Let $\rho _n:= \mathbb {E}\!\left[ \dfrac{X_i(n+1)}{(n+1)\,X_i(n)}\;\bigg |\;\mathcal {F}_n\right]$. Then $\rho _n$ is a strictly deterministic function of *n*, $u(t_n)$, $\lambda _2$, and α, and the process

28 $$\begin{aligned} W_i(n) \;=\; Y_i(n) \prod _{k=\tau _i}^{n-1} \rho _k^{-1} \end{aligned}$$

is a non-negative martingale with respect to the natural filtration $\mathcal {F}_n = \sigma (X(1), \dots , X(n))$. Furthermore, $W_i(n) \rightarrow W_i^{(\infty )}$ almost surely as $n \rightarrow \infty$, and consequently the relative mass fraction $Y_i(n)$ converges almost surely to a non-degenerate limit $Y_i^{(\infty )} \in [0,1]$.

#### Proof

We evaluate the conditional expectation of the absolute lesion size $X_i(n+1)$ given the historical filtration $\mathcal {F}_n$. Conditional on $\mathcal {F}_n$, the next cell joins lesion *i* with probability $\Pi _i(n)$ from Equation (26), so:

29 $$\begin{aligned} \mathbb {E}[X_i(n+1) \mid \mathcal {F}_n]&= X_i(n) + 1 \cdot \Pi _i(n) \nonumber \\&= X_i(n) + \frac{X_i(n)}{\lambda _2 n + u(t_n)} \left[ \lambda _2 + \frac{u(t_n)}{n + \alpha } \right] \nonumber \\&= X_i(n) \left[ 1 + \frac{\lambda _2}{\lambda _2 n + u(t_n)} + \frac{u(t_n)}{(\lambda _2 n + u(t_n))(n + \alpha )} \right] . \end{aligned}$$

Dividing by (n+1) and writing $Y_i(n) = X_i(n)/n$, we obtain

30 $$\begin{aligned} \mathbb {E}[Y_i(n+1) \mid \mathcal {F}_n] \;&=\; \frac{n}{n+1}\,Y_i(n)\!\left[ 1 + \frac{\lambda _2}{\lambda _2 n + u(t_n)} + \frac{u(t_n)}{(\lambda _2 n + u(t_n))(n + \alpha )} \right] \nonumber \\ \;&=\; \rho _n\,Y_i(n), \end{aligned}$$

where the deterministic factor $\rho _n$ admits the explicit form

31 $$\begin{aligned} \rho _n \;=\; \frac{n}{n+1}\left[ 1 + \frac{\lambda _2}{\lambda _2 n + u(t_n)} + \frac{u(t_n)}{(\lambda _2 n + u(t_n))(n+\alpha )}\right] . \end{aligned}$$

In particular $\rho _n$ depends only on *n*, $u(t_n)$, $\lambda _2$, and α, not on the sample path. By construction $\mathbb {E}[W_i(n+1) \mid \mathcal {F}_n] = W_i(n)$, so $W_i(n)$ is a non-negative martingale.

A direct calculation shows that at α=0 the bracket collapses,

$$ 1 + \frac{\lambda _2}{\lambda _2 n + u(t_n)} + \frac{u(t_n)}{n(\lambda _2 n + u(t_n))} \;=\; 1 + \frac{1}{n} \;=\; \frac{n+1}{n}, $$

and hence $\rho _n \big |_{\alpha =0} = 1$ identically. Treating α>0 as a perturbation, expansion of (31) in α/n yields

32 $$\begin{aligned} \rho _n \;=\; 1 - \frac{\alpha \,u(t_n)}{n(n+1)(\lambda _2 n + u(t_n))} + O\!\left( \frac{\alpha ^2}{n^3}\right) . \end{aligned}$$

Hence $$\log \rho _n = -\alpha \,u(t_n)/\bigl (n(n+1)(\lambda _2 n + u(t_n))\bigr ) + O(\alpha ^2/n^3)$$.

By Lemma 1, on the non-extinction event $\Omega ^*$ we have $u(t_n) \asymp n^{\lambda _1/\lambda _2}$ almost surely with random but a.s. finite constants. Substituting the upper bound into (32), the leading correction is of order $n^{\lambda _1/\lambda _2 - 3}$, which is absolutely summable since $\lambda _1/\lambda _2 < 1$. The random prefactor does not affect convergence, as a finite constant times a convergent series remains convergent. Therefore $\prod _{k=\tau _i}^{\infty } \rho _k$ converges almost surely to a strictly positive (random) limit, and the compensating product in (28) is bounded above and below by strictly positive constants for all *n* on $\Omega ^*$. Since $W_i(n)$ is a non-negative martingale bounded in $L^1$ (because $Y_i(n) \in [0,1]$), Doob’s Martingale Convergence Theorem gives $W_i(n) \rightarrow W_i^{(\infty )}$ almost surely. Combined with the boundedness of the compensator,

$$ \Lambda _i(n) \;:=\; \prod _{k=\tau _i}^{n-1} \rho _k, $$

this implies that $Y_i(n)$ itself converges almost surely to a limit $Y_i^{(\infty )} \in [0,1]$. □

#### Remark 1

At leading order ($n \gg \alpha$), $\rho _n \equiv 1$, so $Y_i(n)$ is itself an asymptotic martingale: the relative mass fraction of each lesion stabilizes without correction. The Polya-urn perspective accounts for the small $O(\alpha /n^2)$ correction, which is summable precisely because the migration term $u(t_n)$ grows sub-linearly under the Durrett asymmetry $\lambda _2 > \lambda _1$. We note that non-degeneracy of $Y_i^{(\infty )}$ (i.e., $P(Y_i^{(\infty )}> 0) > 0$) follows from standard arguments for reinforced urns: conditional on the lesion forming at step $\tau _i$, the lesion subsequently accumulates cells at a positive expected rate, and a Borel–Cantelli argument analogous to Corollary 1 prevents extinction of the relative share. A fully rigorous treatment of non-degeneracy and the distribution of $Y_i^{(\infty )}$ is beyond the scope of this paper and is left for future work.

This represents a crucial stochastic insight. The sample paths of individual lesions do not fluctuate chaotically indefinitely. Instead, they stabilize to fixed proportional sizes. If a tumor achieves a 60% mass fraction early in the disease progression due to pure stochastic chance, the martingale convergence guarantees that the fraction will converge almost surely to a limit, and that this limit will lie close to the value frozen in by early stochastic events. The macro-level heterogeneity of the tumor landscape is thus dictated by microscopic, early-stage stochastic fluctuations, with the asymmetry $\lambda _2 > \lambda _1$ ensuring that the migration-driven mixing necessary to redistribute mass dies out before it can erase those initial advantages.

### The Finiteness of Total Lesions via Borel-Cantelli

Finally, we can provide the exact probability theory proof of Theorem 1 without relying on mean-field equations.

#### Corollary 1

(Finiteness of Lesions). Let *L*(*n*) denote the total number of distinct metastatic lesions formed up to step *n*. If $\lambda _2 > \lambda _1$, then $P\left( \lim _{n \rightarrow \infty } L(n) < \infty \right) = 1$.

#### Proof

Let the indicator variable $I_n$ take the value 1 if a new lesion is seeded at step *n*, and 0 otherwise. Thus, $L(n) = \sum _{k=1}^n I_k$. The probability of this event, conditional on the urn history $\mathcal {F}_{n-1}$, is precisely $\Pi _{\text {new}}(n)$, and hence $\mathbb {E}[I_n] = \mathbb {E}[\Pi _{\text {new}}(n)]$.

By Lemma 1, on the non-extinction event $\Omega ^*$ the migration term obeys $u(t_n) \le C\, n^{\lambda _1/\lambda _2}$ almost surely for all $n \ge n_0$, with *C* a.s. finite. Bounding the new-color probability accordingly, the conditional expectation of the indicator scales as:

33 $$\begin{aligned} \mathbb {E}[I_n \mid \mathcal {F}_{n-1}]&= \Pi _{\text {new}}(n) = \frac{\alpha u(t_n)}{(n+\alpha )(\lambda _2 n + u(t_n))} \le \frac{\alpha C\, n^{\lambda _1/\lambda _2}}{\lambda _2 n^2}\,(1 + o(1)) \nonumber \\&= \mathcal {O}\!\left( n^{\frac{\lambda _1}{\lambda _2} - 2} \right) \quad \text {a.s.} \end{aligned}$$

To determine the boundedness of the total lesion count, we sum these bounds over the infinite horizon:

34 $$\begin{aligned} \sum _{n=1}^{\infty } \mathbb {E}[I_n \mid \mathcal {F}_{n-1}] \le \sum _{n=1}^{\infty } \mathcal {O}\!\left( n^{\frac{\lambda _1}{\lambda _2} - 2} \right) \quad \text {a.s. on } \Omega ^*. \end{aligned}$$

Since the primary tumor growth rate is strictly less than the metastatic rate ($\lambda _2> \lambda _1 > 0$), the exponent satisfies:

35 $$\begin{aligned} \frac{\lambda _1}{\lambda _2} - 2 < 1 - 2 = -1. \end{aligned}$$

Because the exponent is strictly less than -1, the infinite summation in Equation (34) constitutes a convergent *p*-series (the random prefactor *C* does not affect convergence), so $\sum _{n=1}^\infty \mathbb {E}[I_n \mid \mathcal {F}_{n-1}] < \infty$ almost surely on $\Omega ^*$. By the conditional Borel–Cantelli Lemma (Lévy’s extension; see Williams, 1991, §12.15), which applies to the dependent indicators $I_n$ precisely because the sum of conditional probabilities converges almost surely, we conclude that $P(I_n = 1 \text { infinitely often} \mid \Omega ^*) = 0$. Consequently, the sequence of de novo seeding events almost surely terminates, and the urn “freezes,” forcing $L(n) \rightarrow L(\infty ) < \infty$ almost surely on the non-extinction event. □

### Technical Caveats and Regularity Assumptions

We would like to make several technical comments.

**(i) Non-degeneracy of** $Y_i^{(\infty )}$ **.** Theorem 2 establishes that the relative mass fraction $Y_i(n)$ converges almost surely to a limit $Y_i^{(\infty )} \in [0,1]$ via Doob’s Martingale Convergence Theorem. Doob’s theorem alone does *not* preclude the degenerate possibility $Y_i^{(\infty )} = 0$ almost surely, which would correspond to the share of lesion *i* being diluted away by incoming cells. For classical Polya-urn schemes with constant migration, non-degeneracy of the limiting proportions is a well-known consequence of exchangeability and de Finetti-type arguments (Pitman 2006), and for time-inhomogeneous reinforced urns it can typically be obtained by combining a Kakutani-type product convergence with a uniform lower bound on the per-step retention probability. In our setting, once lesion *i* has accumulated $X_i(\tau _i) \ge 1$ cells, the probability that it is selected on subsequent steps is bounded below by $\lambda _2 X_i(n)/(\lambda _2 n + u(t_n)) \ge \lambda _2 X_i(n)/((\lambda _2 + C)n)$ for some constant *C* depending on the eventual asymptotics of $u(t_n)$, which is sufficient to ensure that the conditional growth rate of $X_i(n)$ matches the growth rate of *n* to leading order. A fully rigorous proof of $P(Y_i^{(\infty )} > 0 \mid \tau _i < \infty ) = 1$ follows the same outline as the analogous result for generalized Polya urns (Janson 2004) and is omitted.

**(ii) Regularity of the fluid-limit drift.** The infinitesimal drift in Equation (40) contains the ratio $\bar{u}(t)\bar{X}_i(t)/\bar{M}(t)$, which fails to be Lipschitz continuous on the boundary $\{\bar{M} = 0\}$. Kurtz’s theorem in its standard form (Ethier and Kurtz 1986, Chapter 11) requires the drift field $\beta _i(\bar{\textbf{X}}, t)$ to be Lipschitz on a neighborhood of the trajectory. We resolve this in the natural way: the fluid limit is applied on the time interval $[t_0, T]$ for any $t_0 > 0$ chosen after at least one lesion has been established, so that $\bar{M}(t_0) > 0$ deterministically in the fluid regime. On compact subsets of $\{\bar{M} \ge m_0\}$ for any $m_0 > 0$, the drift is Lipschitz with constant uniformly bounded in *t* on $[t_0, T]$, and Kurtz’s convergence theorem applies on this restricted state space. Equivalently, one may rewrite the drift as $\lambda _2 \bar{X}_i + \bar{u}(t)\bar{Y}_i$ with $\bar{Y}_i = \bar{X}_i/\bar{M}$, treat $\bar{Y}_i$ as a separate state variable governed by its own (Lipschitz) evolution equation derived from Theorem 2, and avoid the singularity entirely. The deterministic trajectory in Equation (43) for $\bar{M}(t)$ does not depend on this choice. Only the per-lesion partition $\bar{X}_i = \bar{Y}_i \bar{M}$ does.

**(iii) Stochastic versus fluid-scale growth in** ${\textbf {u}}({\textbf {t}}_{\textbf {n}})$ **.** The scaling $u(t_n) \sim n^{\lambda _1/\lambda _2}$ used in Corollary 1 and in the convergence proof of Theorem 2 would, if read as a deterministic identity, amount to substituting the expected growth $u(t) \sim e^{\lambda _1 t}$, $M(t) \sim e^{\lambda _2 t}$ into quantities that are in fact stochastic, since $Z_1(t)$ and *M*(*t*) are supercritical birth-death branching processes. We have avoided this substitution: Lemma 1 establishes the scaling almost surely on the non-extinction event $\Omega ^*$, with random but a.s. finite two-sided constants, by inverting the supercritical martingale limits $e^{-\lambda _1 t}Z_1(t) \rightarrow W_1 > 0$ and $e^{-\lambda _2 t}M(t) \rightarrow W_2 > 0$ (Athreya and Ney 1972; Durrett 2015). Both the Borel–Cantelli argument in Corollary 1 and the summability argument in Theorem 2 are written to use only the finiteness and positivity of these random constants, which is all that the convergence of the relevant series requires. The conclusions therefore hold almost surely on $\Omega ^*$.

## Fluid Limits and Deterministic Hitting Times

While the martingale analysis developed in Sect. 5 successfully dictates the long-term relative allocation profiles of individual lesions, it indexes the system via discrete cell additions *n*. In clinical applications, tracking absolute timelines is vital. We would like to map these structural limits back to the continuous time-domain *t*. To accomplish this, we utilize density-dependent fluid limit frameworks (Ethier and Kurtz 1986; Kurtz 1970), which formalize the transition from purely stochastic Markov jump processes to continuous, smooth ordinary differential systems as the volume parameter scales.

### Conditioning on the Freezing Event

Before scaling, we address a structural obstruction. In its standard form (Ethier and Kurtz 1986, Ch. 11), Kurtz’s theorem applies to density-dependent families on a state space of *fixed* dimension, with rates that are density-dependent and a drift field that is Lipschitz on a neighborhood of the limiting trajectory. Taken as a whole, the process of Sect. 3.4 satisfies none of these requirements: the dimension of $\textbf{X}(t)$ evolves through *L*(*t*), the migration term $u(t) = u_2 Z_1(t)$ is time-inhomogeneous and is driven by an external compartment rather than by the density of the metastatic compartment itself, and the seeding channel (T6b), being *O*(1) rather than *O*(*N*), vanishes under the scaling.

The resolution is supplied by the analysis already completed. Corollary 1 shows that on $\Omega ^*$ the seeding channel fires only finitely often, so there is an almost surely finite random time

36 $$\begin{aligned} T_{\text {freeze}} \;:=\; \sup \{t \ge 0 : \text {a de novo seeding event occurs at time } t\} \;<\; \infty \quad \text {a.s. on } \Omega ^*, \end{aligned}$$

after which $L(t) \equiv L(\infty )$. We therefore condition on the event $\{L(\infty ) = \ell \}$, which has positive probability for each ℓ in the support of L(∞), and apply the fluid limit on the interval $[T_{\text {freeze}}, T]$ rather than on [0, *T*]. On this event and this interval:

(i) the state space is $\mathbb {N}^{\ell }$, of *fixed* dimension ℓ, so the dimensionality obstruction is removed;
(ii) the seeding channel has genuinely terminated. It is not merely invisible at leading order in *N*, it no longer fires, so the third obstruction is removed exactly rather than approximately;
(iii) the surviving transitions are (T5), with rate $\lambda _2 X_i$, and (T6a), with rate $u(t) X_i/(M+\alpha )$, both of which are density-dependent in $\textbf{X}$ up to the externally driven factor *u*(*t*). The residual time-inhomogeneity is a deterministic, bounded, continuously differentiable multiplier on a compact interval, which the time-inhomogeneous form of the theorem accommodates.

The Lipschitz requirement is handled separately, in caveat (ii) of Sect. 5.4, by restricting to $\{\bar{M} \ge m_0\}$ for some $m_0>0$. This is harmless because $\bar{M}(T_{\text {freeze}}) > 0$ on $\Omega ^*$ by construction.

### Formalization of Kurtz Scaling

We define a macroscopic reference parameter $N \in \mathbb {N}$, which represents a characteristic cellular capacity or volume threshold. We scale our stochastic state vector $\textbf{X}(t)$ into an intensive-variable state vector $\bar{\textbf{X}}^N(t)$, where individual elements represent normalized tumor masses:

37 $$\begin{aligned} \bar{X}_i^N(t) = \frac{X_i(t)}{N}, \quad \bar{M}^N(t) = \frac{M(t)}{N}, \quad \bar{u}^N(t) = \frac{u(t)}{N} = \frac{u_2 Z_1(t)}{N}. \end{aligned}$$

The underlying jump process transitions via discrete increments of size ±1/N. For each established tumor *i*, the transition step is $\textbf{e}_i/N$ with an intensive propensity function. According to Kurtz’s Theorem, if the propensity functions converge uniformly to a Lipschitz-continuous drift field, then over any finite time interval $[T_{\text {freeze}}, T]$ and conditionally on $\{L(\infty )=\ell \}$, the scaled stochastic paths converge almost surely to the deterministic solution of the limiting differential equations as $N \rightarrow \infty$:

38 $$\begin{aligned} \lim _{N \rightarrow \infty } \sup _{0 \le t \le T} \left| \bar{X}_i^N(t) - \bar{X}_i(t) \right| = 0 \quad \text {a.s.} \end{aligned}$$

Applying this scaling to our non-homogeneous continuous-time CRP formulation, the infinitesimal drift $\beta _i(\bar{\textbf{X}}, t)$ for the *i*-th lesion is evaluated by isolating the intensive components:

39 $$\begin{aligned} \beta _i(\bar{\textbf{X}}, t) = \lim _{N \rightarrow \infty } \left[ \lambda _2 \bar{X}_i^N(t) + \bar{u}^N(t) \frac{\bar{X}_i^N(t)}{\bar{M}^N(t) + \frac{\alpha }{N}} \right] = \lambda _2 \bar{X}_i(t) + \bar{u}(t) \frac{\bar{X}_i(t)}{\bar{M}(t)}. \end{aligned}$$

Note that the concentration parameter α enters as α/N in the scaled denominator and is therefore eliminated in the fluid limit. This is the deeper reason why the lesion *count* *L* remains an *O*(1) quantity (see Theorem 1) even as the individual lesion *masses* scale with *N*: *de novo* seeding events are not visible to leading order in the fluid description. Here $\bar{u}(t) = \bar{u}_2 e^{\lambda _1 t}$ describes the continuous, deterministic arrival stream of cells originating from the primary and peritoneal compartments under standard fluid assumptions. This yields a system of coupled non-linear differential equations representing the fluid limit:

40 $$\begin{aligned} \frac{d\bar{X}_i(t)}{dt} = \bar{X}_i(t) \left[ \lambda _2 + \frac{\bar{u}(t)}{\bar{M}(t)} \right] . \end{aligned}$$

### Closed-Form Trajectory Integration

To solve the system of equations defined in Equation (40), we first decouple the total metastatic mass trajectory $\bar{M}(t)$. Summing the derivatives across all *L* lesions, we obtain a linear non-homogeneous ordinary differential equation for the total aggregate burden:

41 $$\begin{aligned} \frac{d\bar{M}(t)}{dt} = \sum _{i=1}^{L} \frac{d\bar{X}_i(t)}{dt} = \lambda _2 \bar{M}(t) + \bar{u}_2 e^{\lambda _1 t}. \end{aligned}$$

We solve this via an integrating factor $\mu (t) = e^{-\lambda _2 t}$:

42 $$\begin{aligned} \frac{d}{dt} \left[ \bar{M}(t) e^{-\lambda _2 t} \right] = \bar{u}_2 e^{(\lambda _1 - \lambda _2)t}. \end{aligned}$$

Integrating from 0 to *t* yields the exact deterministic total mass trajectory:

43 $$\begin{aligned} \bar{M}(t) = M_0 e^{\lambda _2 t} + \frac{\bar{u}_2}{\lambda _1 - \lambda _2} \left( e^{\lambda _1 t} - e^{\lambda _2 t} \right) , \end{aligned}$$

where $M_0 = \bar{M}(0)$ represents the aggregate seed mass at the initiation of the fluid regime.

### Lesion-Level–Corrected Hitting Times

In the baseline Danesh et al. framework, the window of opportunity terminates at a fixed time $T_2$, dictated purely by the total aggregate mass crossing a lethal burden: $M(T_2) = 10^9$ cells. However, clinical detection modalities such as transvaginal ultrasound or computed tomography (CT) are fundamentally constrained by physical spatial resolution. They do not measure distributed individual cells, but rather they detect localized density masses. Consequently, radiological detectability is a threshold function of the *largest individual lesion*, $X_{\max }(t) = \max _i X_i(t)$.

Using our fluid limit derivation, we denote $Y_{\max } = \max _i Y_i^{(\infty )}$ as the almost-sure limiting mass fraction of the dominant tumor sink established in the stochastic phase (cf. Theorem 2). Once the early stochastic ratios have stabilized, the trajectory of the largest tumor is well-approximated by $X_{\max }(t) \approx Y_{\max }\,M(t)$. We define our lesion-level–corrected clinical screening window endpoint $\tau _{\text {detect}}$ as the hitting time:

44 $$\begin{aligned} \tau _{\text {detect}} = \inf \left\{ t > 0 : Y_{\max } M(t) \ge C_{\text {detect}} \right\} , \end{aligned}$$

where $C_{\text {detect}}$ represents the minimum cellular resolution limit of standard abdominal imaging.

In the late-stage disease where *t* is large, the aggregate mass in Equation (43) is dominated by the pure metastatic growth exponent $e^{\lambda _2 t}$ since $\lambda _2 > \lambda _1$. Specifically, the migration contribution $\bar{u}_2(e^{\lambda _1 t} - e^{\lambda _2 t})/(\lambda _1-\lambda _2)$ has the same sign as $M_0 e^{\lambda _2 t}$ (both positive, since $\lambda _1-\lambda _2 < 0$), but its growth is bounded by $\bar{u}_2 e^{\lambda _2 t}/(\lambda _2-\lambda _1)$, a fixed multiple of the dominant exponential. For $M_0$ on the order of $10^5$ cells or larger, this migration correction contributes at most an *O*(1) multiplicative shift to the asymptotics, and may be absorbed into an effective initial mass without changing the leading-order hitting-time scaling. Dropping the transient migration terms, we isolate $\tau _{\text {detect}}$ algebraically:

45 $$\begin{aligned} Y_{\max } M_0 e^{\lambda _2 \tau _{\text {detect}}} \approx C_{\text {detect}} \implies \tau _{\text {detect}} \approx \frac{1}{\lambda _2} \ln \left( \frac{C_{\text {detect}}}{M_0 Y_{\max }} \right) . \end{aligned}$$

**Fig. 2:**
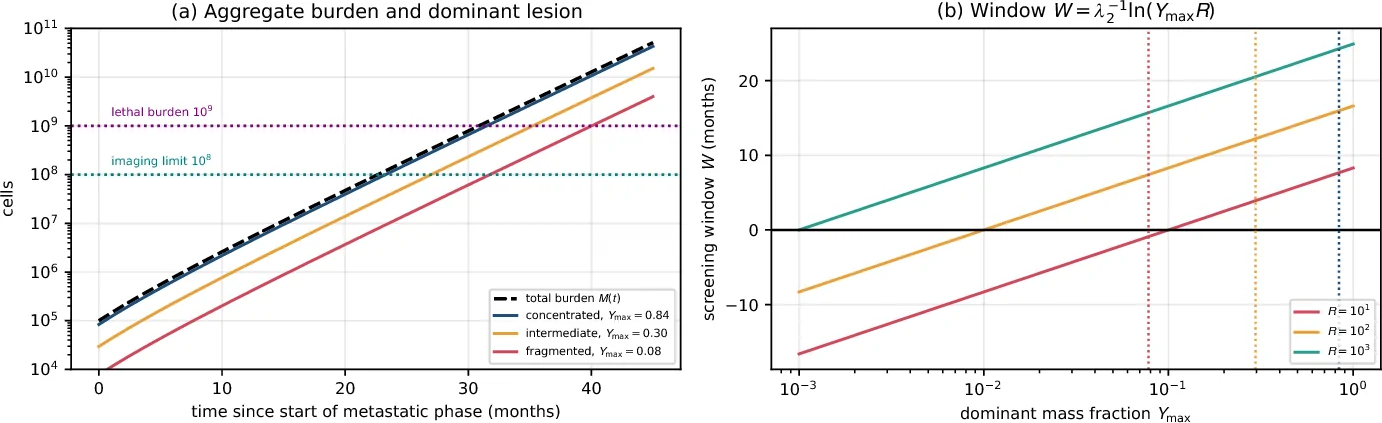
Fluid-limit trajectories and the detectability criterion. **a** Total metastatic mass *M*(*t*) (dashed) together with the dominant lesion $X_{\max }(t) = Y_{\max }M(t)$, drawn at three representative values of the dominant mass fraction spanning the range the model produces. As $Y_{\max }$ falls, the dominant lesion crosses the imaging limit progressively later relative to the lethal burden, compressing the window. **b** The window $W = \lambda _2^{-1}\ln (Y_{\max }R)$ of Equation (46) against $Y_{\max }$, for three values of the detection-to-lethality ratio *R*. Dotted verticals mark the three cases of panel (a). The window closes (W<0) only for the smallest mass fraction shown and only at R=10, which is the basis for reporting compression rather than categorical invisibility. The values of $Y_{\max }$ actually attained, and the parameters that produce them, are established in Sect. 7.

Figure 2 illustrates the deterministic trajectories derived from the fluid limit, mapping the total metastatic mass *M*(*t*) alongside the dominant lesion $X_{\max }(t)$ over a 45-month horizon, together with the window that follows from them.

Equation (45) has a consequence worth isolating, because it is both sharper and more robust than the trajectory comparison it summarizes. Writing $\tau _{\text {lethal}} = \lambda _2^{-1}\ln (M_{\text {lethal}}/M_0)$ for the time at which the aggregate burden becomes lethal, the screening window is

46 $$\begin{aligned} W \;=\; \tau _{\text {lethal}} - \tau _{\text {detect}} \;=\; \frac{1}{\lambda _2}\,\ln \!\left( \frac{Y_{\max }\,M_{\text {lethal}}}{C_{\text {detect}}}\right) , \end{aligned}$$

which is *independent of* $M_0$ : the seed mass cancels. Since *W* is a difference of two hitting times it may be negative, and the sign carries the clinical content. A positive *W* is the interval during which the dominant lesion is radiologically visible while the aggregate burden is still sub-lethal, which is the window in the usual sense. A negative *W* means that the ordering of the two events is reversed: the dominant lesion reaches the resolution limit only after the aggregate burden has already passed the lethal threshold, so that the window never opens at all. This is a statement about resolution-limited imaging and not about detectability in general, since a serum marker tracking the aggregate mass *M*(*t*) is unaffected by how that mass is partitioned among lesions. Writing $R:= M_{\text {lethal}}/C_{\text {detect}}$ for the detection-to-lethality ratio, we obtain a parameter-free detectability criterion,

47 $$\begin{aligned} W> 0 \quad \Longleftrightarrow \quad Y_{\max } \;>\; \frac{C_{\text {detect}}}{M_{\text {lethal}}} \;=\; \frac{1}{R}. \end{aligned}$$

The disease is radiologically detectable before it is lethal precisely when the dominant lesion holds more than a fraction 1/*R* of the total metastatic mass. With the Danesh et al. thresholds $M_{\text {lethal}} = 10^9$ and $C_{\text {detect}} = 10^8$ one has R=10 and the criterion reads $Y_{\max } > 0.1$.

We flag immediately that the numerical value of the threshold is sensitive to *R*, which is the least well determined quantity in the framework. The value R=10 inherits the Danesh et al. lethal burden of $10^9$ cells, but advanced ovarian cancer routinely presents at debulking with considerably more disease than one cubic centimeter, and the calibration of Sect. 8 independently infers a burden of order $10^{10}$ cells at surgery. Section 7.6 therefore reports the window across $R \in \{10, 10^2, 10^3, 10^4\}$. The qualitative conclusion, that the window contracts monotonically as $Y_{\max }$ falls, is independent of *R*. The stronger claim that the window *closes entirely* holds only at the smallest values of *R*, and we do not rely on it.

Panel (a) of Fig. 2 shows the consequence for the trajectories. When the dominant lesion holds most of the mass it crosses the imaging threshold well before the aggregate reaches the lethal burden, and the window is comfortably open. As the mass fraction falls the crossing moves progressively later relative to the lethal burden, until for the smallest fraction shown the two nearly coincide. Panel (b) plots the window itself against $Y_{\max }$ and makes the role of *R* explicit: the curves are parallel in $\ln Y_{\max }$ and shift upward with *R*, so that the value of *R* determines where, and whether, the window closes. Section 8 shows that the mass fractions attained at clinically calibrated parameters are not small enough to drive *W* below zero. The model therefore predicts that fragmentation shortens the window substantially, from roughly 7.6 to 2.9 months across the range of lesion counts observed clinically, without closing it.

## Stochastic Simulation Study

This section reports a systematic comparison between direct stochastic simulation and the analytic predictions of Sects. 4–6. We validate the freezing theorem quantitatively, verify the martingale stabilization of mass shares, measure the rate of convergence to the fluid limit, and map the dependence of the phenotype on the model parameters.

### Simulation Method

We simulate the process of Sect. 3.4 exactly, without tau-leaping or other time discretization. Between successive events in the metastatic compartment the mass *M* is constant while the migration intensity $u(t) = u_0 e^{\lambda _1 t}$ varies smoothly, so the cumulative hazard admits the closed form

48 $$\begin{aligned} H(\sigma ) \;=\; \lambda _2 M \sigma \;+\; \frac{u_0}{\lambda _1}\,e^{\lambda _1 t}\left( e^{\lambda _1 \sigma } - 1\right) , \end{aligned}$$

and inter-event times are obtained by drawing $E \sim \textrm{Exp}(1)$ and solving H(σ)=E by Newton iteration. Size-biased selection of a lesion is performed in *O*(1) by maintaining a cell-to-lesion index array and drawing a uniform position, so that a realization is linear in the number of events. Unless stated otherwise, runs use the Danesh et al. rates $\lambda _1 = 0.173$, $\lambda _2 = 0.277\ \text {mo}^{-1}$ and terminate at $M = 10^5$ cells.

### Quantitative Validation of the Freezing Theorem

Theorem 1 predicts that the expected number of lesions is finite and equal to $\mathbb {E}[L(\infty )] = 1 + \int _0^\infty r_{\text {new}}(t)\,dt$. Table 2 compares this integral, evaluated on the fluid trajectory (43), against the mean of 200 independent realizations at each α.

**Table 2:** Validation of Theorem 1. Ensemble of 200 realizations per α at $u_0 = 1$, run to $M = 10^5$. Agreement is within sampling error across a forty-fold range in *L*. The $Y_{\max }$ column is the ensemble mean of the terminal dominant mass fraction $\max _i X_i(M)/M$. The *W* column is the ensemble mean of the per-realization window $W = \lambda _2^{-1}\ln (Y_{\max } R)$ from Equation (46), evaluated at R=10. Because *W* is a concave function of $Y_{\max }$, this mean is not the window of the mean fraction, and the two differ slightly

α	*L* (simulated)	$\mathbb {E}[L(\infty )]$ (theory)	rel. error	$Y_{\max }$	*W* (months)
0.5	2.24±1.08	2.06	+8.8%	0.838±0.184	7.56±0.93
2	4.89±1.77	4.47	+9.4%	0.652±0.201	6.57±1.19
10	12.96±3.35	12.43	+4.3%	0.438±0.147	5.13±1.21
50	33.96±6.78	34.20	-0.7%	0.296±0.101	3.72±1.16
200	76.88±13.22	80.66	-4.7%	0.242±0.097	2.93±1.35

**Fig. 3:**
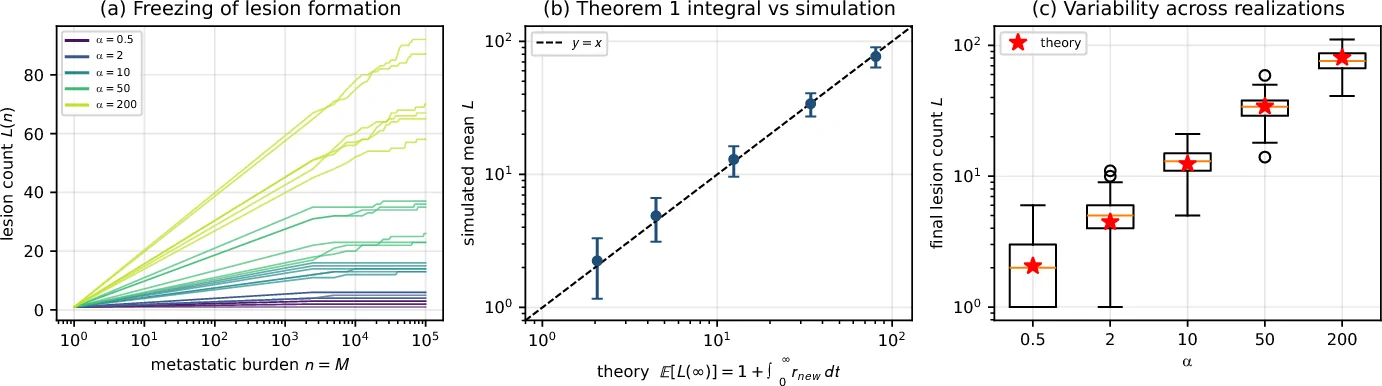
Freezing of lesion formation. **a** Trajectories *L*(*n*) for twelve realizations at each α. The count saturates, with the required horizon growing with α. **b** Simulated mean lesion count against the theoretical integral $\mathbb {E}[L(\infty )]$, with the identity line. Agreement holds across a forty-fold range. **c** Ensemble distribution of the final lesion count, with the theoretical prediction marked

Fig. 3 displays the same comparison graphically. Panel (b) plots the simulated mean against the theoretical integral on logarithmic axes together with the identity line, and the points lie on that line across the full range, which is the visual statement of Table 2. Panel (c) shows the ensemble distribution of the final lesion count as a box plot with the theoretical value overlaid, and makes clear that the agreement holds not only in the mean but in the bulk of the distribution: the theoretical prediction falls near the median at every α, while the spread grows with α roughly as $\sqrt{\mathbb {E}[L]}$, as would be expected of a count governed by an inhomogeneous Poisson mechanism. Panel (a) shows the underlying trajectories *L*(*n*), which saturate for $\alpha \le 50$. We note explicitly that at α=200 the lesion count is still rising at the end of the simulated horizon. This is a statement about time scales, not a failure of the theorem: freezing is asymptotic, and the burden at which the final seeding event occurs grows steeply with α. Recording that burden directly over 25–30 realizations per value of α, run to $M = 3 \times 10^5$, the median rises from n=2 at α=0.5 to n=84 at α=2, $n = 1.0 \times 10^4$ at α=10, $n = 1.5 \times 10^5$ at α=50 and $n = 2.0 \times 10^5$ at α=200, five orders of magnitude across the range considered.

**Fig. 4:**
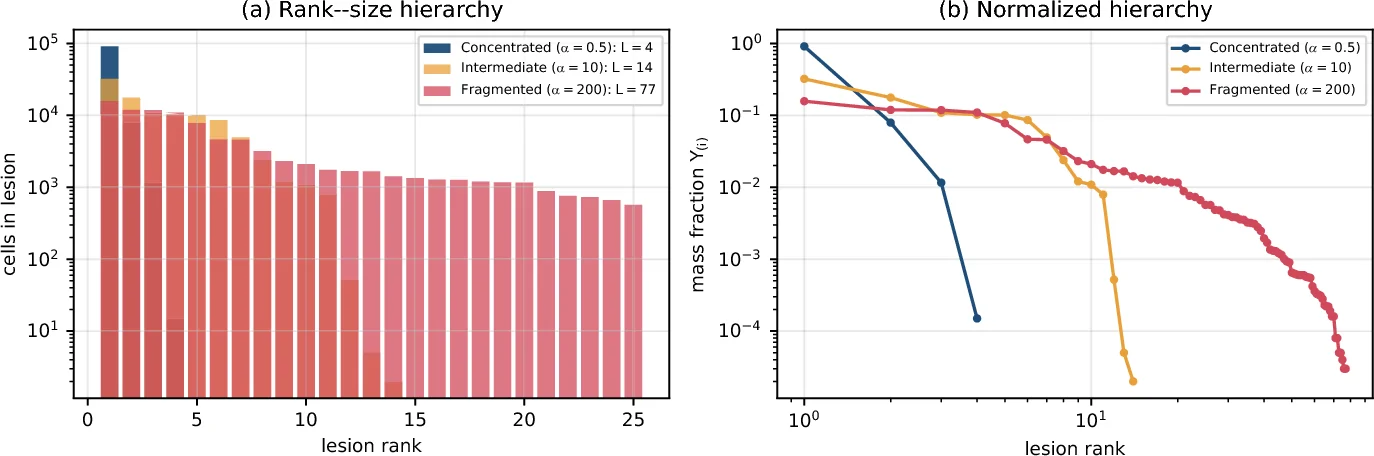
Lesion-size hierarchy at $M = 10^5$ for three values of α at $u_0 = 1$. **a** Rank–size distribution, with the lesion count realized in each run. **b** The same hierarchies normalized to mass fractions $Y_{(i)} = X_{(i)}/M$, with rank on a logarithmic axis. Each panel shows a *single* realization rather than an ensemble, so the counts in the legend (L=4, 14, 77) are individual draws and are not expected to equal the ensemble means of Table 2 (2.24, 12.96, 76.88). The standard deviations reported there indicate how much run-to-run variation to expect. These panels are shown to convey the shape of the size hierarchy, which ensemble means do not capture

### Martingale Stabilization of Mass Shares

**Fig. 5:**
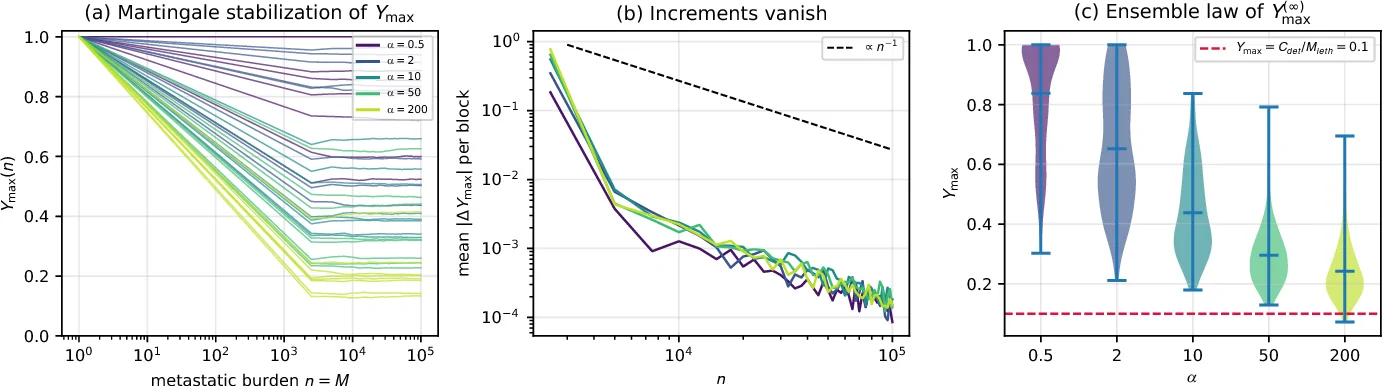
Stabilization of relative mass shares, colored by α as in the legend of panel (a). **a** $Y_{\max }(n)$ along twelve individual realizations per α, flattening after $n \approx 10^3$–$10^4$. **b** Mean absolute change in $Y_{\max }$ between consecutive recorded snapshots, averaged over those realizations, decaying faster than the reference slope $n^{-1}$. **c** Ensemble law of the limit $Y_{\max }^{(\infty )}$ over 200 realizations per α, drawn as violins: the shaded body is a kernel density estimate of the distribution, its width at a given height being proportional to the density of realizations attaining that value, and the horizontal bars mark the extremes and the mean. The dashed line marks the detectability threshold $Y_{\max } = 1/R = 0.1$ of Equation (47). At every α tested the bulk of the distribution lies above it

Theorem 2 predicts that $Y_i(n)$ converges almost surely. Figure 5a shows $Y_{\max }(n)$ flattening after $n \approx 10^3$–$10^4$, and panel (b) confirms that the mean absolute increment per recorded block decays faster than $n^{-1}$.

The consequence of this stabilization for the shape of the disease is shown in Fig. 4. At α=0.5 a single lesion holds most of the mass and the rank–size profile falls away sharply after the first one or two entries. At α=200 the profile is far flatter, with mass spread over tens of lesions and no single dominant sink. Panel (b) makes the contrast scale-free by normalizing to mass fractions: the concentrated case is a short, steep sequence, whereas the fragmented case decays slowly over more than an order of magnitude in rank. These are the two regimes whose clinical consequences Sect. 8 quantifies.

Returning to Fig. 5, panel (c) reports the ensemble law of the limit. The spread is substantial. At α=0.5 the standard deviation of $Y_{\max }$ is 0.18 on a mean of 0.84. This dispersion is itself a prediction of the theory rather than simulation noise: Theorem 2 asserts that the limit is a genuinely random variable fixed by early events, so a single realization per parameter value would misrepresent the model. Across the ensemble, $Y_{\max }$ remains bounded away from zero at every α tested, which is consistent with the non-degeneracy asserted in caveat (i) of Sect. 5.4 but not proved there.

### Convergence to the Fluid Limit

Sect. 6.1 argued that conditioning on the freezing event places the system inside the standard density-dependent framework. We verify this numerically. Starting from a scaled initial condition $\textbf{X}^N(0) = N\textbf{x}_0$ after freezing, with $L(\infty ) = \ell = 5$ held fixed, we measure the supremum relative deviation of the rescaled paths from the deterministic solution. Table 3 reports the result for both the aggregate mass and the individual lesion trajectories, together with the local log-log slopes between consecutive system sizes.

**Table 3:** Convergence to the fluid limit, conditional on $\{L(\infty )=5\}$ and restricted to $[T_{\text {freeze}},T]$. Local log-log slopes are reported between consecutive rows

*N*	sup. rel. error (total)	slope	sup. rel. error (per-lesion)	slope
20	0.1923±0.0834	n/a	0.6592±0.2787	n/a
100	0.0777±0.0317	-0.563	0.2920±0.0859	-0.506
500	0.0395±0.0138	-0.420	0.1428±0.0473	-0.445
2000	0.0157±0.0043	-0.666	0.0633±0.0191	-0.586
10000	0.0110±0.0020	-0.223	0.0229±0.0038	-0.632

Both the aggregate and the individual lesion trajectories converge at a rate consistent with the $N^{-1/2}$ predicted by the functional central limit theorem accompanying Kurtz’s law of large numbers. The per-lesion result is the one that matters for the present application: it confirms that each lesion tracks $\bar{X}_i(t) = \bar{Y}_i(0)\bar{M}(t)$, which is precisely the frozen-share structure asserted by Theorem 2, and it justifies the use of $X_{\max }(t) \approx Y_{\max } M(t)$ in Sect. 6.

### The Phenotype is Governed by Two Parameters, Not One

The analysis so far has emphasized α. The simulations show that this is incomplete. Table 4 reports the mean $Y_{\max }$ over a 7×9 grid in the migration intensity $u_0$ and the concentration parameter α, logarithmically spaced in both, with 30 realizations per cell.

**Table 4:** Mean $Y_{\max }$ over the $(\alpha , u_0)$ grid, 30 realizations per cell at $M = 10^5$. At low migration intensity the dominant fraction remains high even for very large α, while at high migration intensity it falls below the detectability threshold 1/R=0.1

$u_0 \backslash \, \alpha$	0.5	1.5	5	15	50	150	500	1000	2000
0.05	0.985	0.940	0.851	0.782	0.718	0.759	0.722	0.667	0.685
0.2	0.940	0.765	0.695	0.617	0.592	0.482	0.433	0.448	0.399
1	0.847	0.676	0.481	0.363	0.294	0.204	0.212	0.186	0.172
3	0.839	0.657	0.422	0.281	0.197	0.162	0.112	0.089	0.094
10	0.818	0.555	0.346	0.225	0.133	0.087	0.063	0.054	0.054
30	0.815	0.609	0.364	0.187	0.108	0.059	0.038	0.032	0.027
100	0.760	0.612	0.322	0.157	0.077	0.040	0.022	0.018	0.013

The dependence on $u_0$ is as strong as the dependence on α. At $u_0 = 0.05$ the dominant lesion retains two thirds of the mass even at α=2000, whereas at $u_0 = 100$ the same α yields $Y_{\max } = 0.013$, a fiftyfold difference produced entirely by the migration intensity. The miliary phenotype is therefore not a consequence of high mesothelial receptivity alone. It requires high receptivity *together with* a substantial flux of cells arriving from the peritoneal compartment. This is a genuine model output rather than a restatement of the parameterization. It is also consistent with the clinical observation of Eng et al. (2017) that miliary disease is accompanied by substantially greater ascites volume: in stage IIIC the reported median was 2000 mL for miliary against 500 mL for non-miliary disease, an average excess of about 710 mL after adjusting for stage. To the extent that ascites volume is a proxy for the peritoneal tumor-cell burden feeding the metastatic compartment, this is the direction the model requires.

**Fig. 6:**
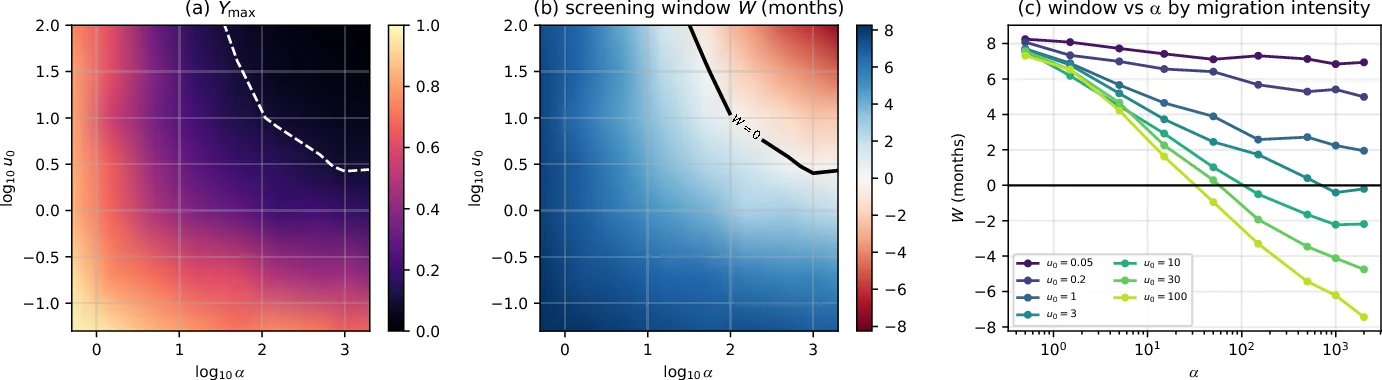
Joint dependence of the phenotype on α and the migration intensity $u_0$. **a** Mean $Y_{\max }$. **b** Screening window *W* from Equation (46), with the W=0 contour marked. **c** *W* against α at each migration intensity.

Applying the criterion (47) to this grid, the boundary W=0 is a curve in the $(\alpha , u_0)$ plane rather than a threshold in α, and is displayed in Fig. 6b. At the ensemble level with $u_0 = 1$, the probability of a non-positive window is zero for $\alpha \le 50$ and 0.015 at α=200, so that even in the fragmented regime the model describes a per-patient failure probability rather than a deterministic phenotype label.

### Sensitivity to the Detection-to-Lethality Ratio

Since the criterion (47) depends on $R = M_{\text {lethal}}/C_{\text {detect}}$, we report the window across four values of *R*. Counting cells of the $(\alpha ,u_0)$ grid at which W<0: at R=10, fifteen of sixty-three cells have a closed window. At $R = 10^2$, $10^3$ and $10^4$, none do. The window itself remains strongly compressed at larger *R*. At $R = 10^2$ it falls from 16.6 months at $(\alpha , u_0) = (0.5, 0.05)$ to 0.9 months at (2000, 100), but it does not close.

We regard the compression, not the closure, as the robust prediction. The closure result requires R=10, which follows from the Danesh et al. lethal burden of $10^9$ cells. The calibration of the next section infers a burden an order of magnitude larger at the time of surgery, and patients evidently survive well beyond $10^9$ cells. We therefore state the clinical conclusion in terms of a contraction of the window that grows with the degree of fragmentation, and we do not claim that imaging is categorically incapable of detecting miliary disease.

### Robustness to Stochastic Migration

The results above treat the migration intensity as the deterministic function $u(t) = u_0 e^{\lambda _1 t}$, which fixes the martingale limit $W_1$ of the peritoneal compartment. Simulating $Z_1$ explicitly as a linear birth–death process instead, so that $u(t) = u_2 Z_1(t)$ carries its own noise, changes nothing detectable: at α=0.5,10,200 the stochastic-$Z_1$ runs give $Y_{\max } = 0.825 \pm 0.203$, 0.413±0.149, 0.239±0.113 against deterministic-migration controls of 0.852±0.173, 0.404±0.160, 0.238±0.092 at matched mean intensity. The urn randomness dominates the migration-process randomness, which justifies the deterministic simplification used elsewhere.

## Calibration Against Clinical Data

The concentration parameter has so far been treated as a free quantity. This section calibrates it against published surgical data and validates the calibration out of sample.

### Calibration Targets

We use three facts from two independent studies. Auer et al. (2015) give an operational definition of the two spread types at primary surgery: miliary disease comprises more than twenty peritoneal implants, most of them smaller than 2 cm, while non-miliary disease comprises no implants or few implants larger than 2 cm. This converts the qualitative phenotype into a threshold on a quantity the model predicts directly, namely L>20. Eng et al. (2017) report the prevalence of the miliary phenotype in a retrospective review of operative records from primary debulking surgery, none of the patients having received neoadjuvant chemotherapy: of 938 eligible patients, 761 records (81%) contained sufficient detail to assess the spread pattern, and among these the miliary phenotype was scored in 249 cases, or 32.7% (Clopper–Pearson 95% CI 29.4–36.0%). The implant-size criterion is held back and used for validation. A 2 cm sphere corresponds to $\tfrac{4}{3}\pi \,\text {cm}^3 \approx 4.19 \times 10^9$ cells at the conventional conversion.

One assumption in combining these sources should be made explicit, since the two studies do not classify patients by the same rule. Auer et al. (2015) apply an explicit count-and-size criterion at surgery, whereas Eng et al. (2017) scored archived operative reports by direct mention of miliary disease or by descriptions of small nodules and plaques matched against reference images, without applying a numerical implant count. Three physician readers, trained on a common set of reference images, scored the records independently, and the frequency with which each assigned the miliary label did not differ significantly between them. The frequency was also stable across the period of the review. The criterion nonetheless remains narrative rather than quantitative, and agreement on marginal rates is weaker evidence than agreement case by case, which the published data do not report. We therefore assume that the two definitions identify the same underlying phenotype, so that the prevalence reported by Eng et al. (2017) may be read as the prevalence of L>20. This assumption is not testable from the published data, and if the narrative criterion is systematically more or less inclusive than the twenty-implant threshold, the fitted α would shift accordingly. Auer et al. (2015) additionally admit a third category for cases of very high tumor load that cannot be assigned to either type, which we do not model.

### Fit

The fit proceeds in two stages. We first obtain the conditional law of *L* given α by direct simulation, then mix that law over a population distribution for α chosen to reproduce the observed prevalence.

**Fig. 7:**
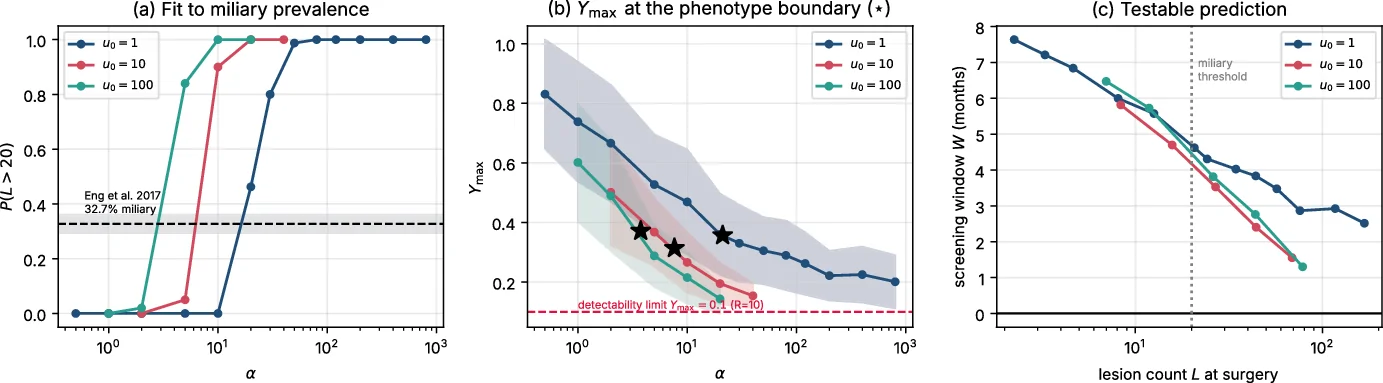
Calibration against clinical series. a P(L>20) against α at three migration intensities, with the Eng et al. (2017) prevalence and its confidence interval. **b** $Y_{\max }$ against α, where stars mark the phenotype boundary $\alpha ^\star$ at each $u_0$, whose ordinate is nearly invariant. **c** The predicted relation between lesion count at surgery and the screening window

The conditional law shows a sharp transition. Simulating 200 realizations at each of thirteen values of α at $u_0 = 1$, the probability P(L>20) is numerically zero for $\alpha \le 10$, rises to 0.463 at α=20 and 0.800 at α=30, and reaches unity by α=80. This curve is the blue trace in Fig. 7a. Interpolating to $P(L>20) = \tfrac{1}{2}$ locates the phenotype threshold at $\alpha ^\star = 21.1$.

For the population stage we model inter-patient variation as $\alpha \sim \textrm{LogNormal}(\mu ,\sigma )$ and solve for μ so that

49 $$\begin{aligned} \int _0^\infty P(L > 20 \mid \alpha )\, f(\alpha ; \mu , \sigma )\, d\alpha \;=\; 0.327 , \end{aligned}$$

the prevalence reported by Eng et al. (2017). A single prevalence figure cannot identify two parameters, so we fix σ and solve for μ, repeating this across the plausible range of σ. Table 5 reports the result. The recovered median α lies between 8.7 and 15.8 across that range, and we report this interval as an identified set rather than a point estimate. The last column gives the median dominant mass fraction implied by each fit.

**Table 5:** Population fit of $\alpha \sim \textrm{LogNormal}(\mu ,\sigma )$ to the miliary prevalence of Eng et al. (2017). For each fixed σ, μ is solved from Equation (49), so the fitted prevalence reproduces the target 32.7% exactly by construction in every row. The recovered median α is stable across the range, varying by less than a factor of two while σ varies fourfold

σ	μ	Median α	Interquartile range of α	Fitted prevalence	Median $Y_{\max }$
0.50	2.763	15.8	11.3–22.2	0.327	0.405
0.75	2.679	14.6	8.8–24.2	0.327	0.420
1.00	2.582	13.2	6.7–26.0	0.327	0.434
1.25	2.480	11.9	5.1–27.8	0.327	0.448
1.50	2.375	10.8	3.9–29.6	0.327	0.461
2.00	2.161	8.7	2.3–33.5	0.327	0.485

### Out-of-Sample Validation

The fit used lesion counts only. We now ask what implant sizes the fitted parameters imply, and compare against the held-out criterion. Writing $X_{\max } = Y_{\max } M_{\text {obs}}$ for the largest lesion at a total burden $M_{\text {obs}}$ and converting to a diameter, the representative non-miliary value α=2 gives $Y_{\max } = 0.666$ and the representative miliary value α=50 gives $Y_{\max } = 0.305$. At $M_{\text {obs}} = 10^{10}$ cells these yield largest-implant diameters of 2.33 cm and 1.80 cm respectively, which satisfy the held-out criterion in both directions simultaneously: the non-miliary implant exceeds 2 cm and the miliary implant does not. At $M_{\text {obs}} = 10^9$ the non-miliary prediction fails, and at $10^{12}$ the miliary prediction fails. Thus $10^{10}$ is the unique order of magnitude in the range tested at which both hold.

We regard this as the substantive answer to the concern that the phenotypes are encoded rather than explained. Nothing in the CRP parameterization compels the count threshold and the size threshold to be mutually consistent at a clinically plausible burden. Similarly, the total burden of roughly 10 g at surgery is implied by the model and not prescribed. It also exposes a tension: the inferred burden at surgery exceeds the Danesh et al. lethal threshold of $10^9$ cells by an order of magnitude, which is the reason we treat *R* as uncertain in Sect. 7.6.

### A Structural Invariant

Repeating the calibration at three migration intensities gives $\alpha ^\star = 21.11$, 7.65 and 3.76 at $u_0 = 1$, 10 and 100. The threshold in α therefore falls by a factor of 5.6 as the migration intensity rises by a factor of 100, confirming that α is not identifiable in isolation. The corresponding values of $Y_{\max }$ at the boundary, however, are 0.357, 0.314 and 0.371, which is nearly invariant.

The clinical miliary/non-miliary distinction thus corresponds, within the model, to a roughly fixed threshold in the dominant mass fraction near $Y_{\max } \approx 0.3$, irrespective of which combination of receptivity and migration produced it. Figure 7 displays the three components of this argument: panel (a) shows the fit of P(L>20) to the observed prevalence, panel (b) shows $Y_{\max }$ against α with the phenotype boundary marked at each migration intensity, so that the near-invariance of its ordinate is visible directly, and panel (c) shows the predicted relation between lesion count and screening window that underlies prediction (P1) below. This is a reinterpretation of an established surgical classification in terms of a quantity the classification does not explicitly measure, and it is not a restatement of the parameterization.

### Predictions

Because $Y_{\max }$ declines monotonically with *L*, and $W = \lambda _2^{-1}\ln (Y_{\max } R)$, the model predicts a monotone decreasing relation between lesion count at surgery and imaging lead time. At $u_0 = 1$ and R=10 the predicted window falls from 7.6 months at L≈2 to 4.6 months at L≈21 and 2.5 months at L≈168. We state four corresponding predictions.

1. *Lesion count predicts imaging lead time.* Among patients with prior cross-sectional imaging, the interval between the first radiologically visible peritoneal lesion and the attainment of a lethal burden should decrease monotonically with the lesion count recorded at debulking.
2. *The phenotype boundary is a mass-fraction threshold.* Classification as miliary versus non-miliary should correspond to the largest-implant mass fraction falling below or above approximately 0.3, a quantity computable from Peritoneal Cancer Index lesion-size scores together with total resected volume.
3. *Migration intensity shifts the boundary.* Patients with a higher peritoneal tumor-cell burden should cross into the miliary phenotype at lower mesothelial receptivity, so that measures of that burden and the implant count should be positively associated at fixed implant size. The association between miliary spread and greater ascites volume reported by Eng et al. (2017) is consistent with this direction, though ascites volume is only an indirect proxy for tumor-cell burden and a direct test would require quantifying cells rather than fluid.
4. *Biomarker–imaging discordance widens with fragmentation.* Since CA-125 tracks the aggregate *M*(*t*) while imaging tracks $Y_{\max } M(t)$, the discordance between a positive biomarker trajectory and a negative scan should increase with lesion count.

We note that (P1)–(P4) are consequences of the framework, not of any particular value of α, and that (P2) in particular does not require estimating α at all.

### What the Calibration Does Not Support

Two distinct thresholds in α must be kept apart here. The first is the phenotype boundary $\alpha ^\star$, at which half of patients exceed twenty implants and are classified as miliary. It takes the values 21.1, 7.65 and 3.76 at $u_0 = 1$, 10 and 100. The second is the invisibility threshold, at which $Y_{\max }$ falls below 1/*R* and the window closes. At R=10 the latter is reached only for $\alpha \gtrsim 200$ at $u_0 = 10$, or $\alpha \gtrsim 50$ at $u_0 = 100$, which is between thirteen and twenty-six times the corresponding $\alpha ^\star$.

The calibrated population sits between the two. Its median α of 8.7 to 15.8 falls modestly below the phenotype boundary $\alpha ^\star = 21.1$ at $u_0 = 1$, by a factor of between 1.3 and 2.4, which is what a miliary prevalence of one third requires. It falls below the invisibility threshold by more than an order of magnitude. The model therefore predicts that most patients meeting the clinical definition of miliary disease should remain detectable in principle, with a short but positive window, and that structural invisibility is confined to the upper tail of the fitted α distribution. Integrating the fitted lognormal above the invisibility threshold puts the affected fraction below 1% at σ=0.5 and at roughly 6 to 19% at σ=2.0, depending on the migration intensity assumed, so the size of that tail is itself poorly determined by the available data. We accordingly frame the clinical conclusion in terms of window compression rather than categorical failure of imaging.

## Biological and Clinical Implications

The structural integration of the Chinese Restaurant Process with continuous-time multi-type branching equations yields clinical insights. By formalizing how cellular division and preferential capture interact, this model provides a mathematical rationale for long-standing empirical observations in gynecological oncology regarding disease presentation, radiological screening trial shortfalls, and surgical constraints.

### Pathophysiology of Miliary Versus Bulky Metastasis

In clinical practice, epithelial ovarian cancer, particularly high-grade serous ovarian cancer (HGSOC), presents along a morphological spectrum defined by the size and distribution of intraperitoneal implants (Bowtell et al. 2015). Our model accounts for this variance through the concentration parameter α. Biologically, α represents the interface between cellular adhesion efficiencies and mesothelial receptivity.

Clinical studies confirm that patients presenting with a miliary phenotype (corresponding to our high-α, high-migration regime) exhibit widely distributed, micronodular disease characterized by numerous sub-millimeter implants blanketing the visceral and parietal surfaces (Auer et al. 2015). Our model provides a candidate mechanism for this presentation: when α is large, the de novo seeding rate $r_{\text {new}}(t)$ remains active for an extended temporal window during early oncogenesis. Sect. 7.5 shows that this requires a substantial migration flux as well as high receptivity. At low migration intensity even very large α fails to fragment the disease. Cells are shed into the peritoneal fluid and implant independently across the abdomen rather than being captured by existing masses, resulting in a flat size distribution where the dominant mass fraction $Y_{\max }$ is minimized.

Conversely, the bulky phenotype (corresponding to our low-α regime) is characterized by a small number of large, vascularized tumor masses, typically isolated on the greater omentum or pelvic wall (Torres et al. 2017). In this regime, our model demonstrates that preferential attachment dominates early, transforming the first few seeded lesions into massive cellular sinks that capture the vast majority of subsequent migrating cells.

### Screening Pitfalls and the Limits of Resolution-Bound Imaging Modalities

The clinical window of opportunity for screening has historically been modeled under the assumption that total tumor volume correlates with detectability (Danesh et al. 2012; Havrilesky et al. 2008). Large-scale clinical screening endeavors, such as the United Kingdom Collaborative Trial of Ovarian Cancer Screening (UKCTOCS) and the Prostate, Lung, Colorectal, and Ovarian (PLCO) Cancer Screening Trial, have evaluated transvaginal ultrasound (TVUS) and CA-125 serum biomarkers to lower mortality, yielding mixed outcomes (Jacobs et al. 2016). Our fluid limit analysis provides a partial and model-conditional explanation for these screening shortfalls.

Imaging modalities like CT, MRI, and TVUS are bounded by physical resolution limits, requiring a localized mass to exceed a critical diameter (typically 1 cm, or $\sim 10^8$ cells) to be classified as positive. In a patient with a fragmented phenotype the dominant mass fraction $Y_{\max }$ is depressed, and by Equation (46) the screening window contracts logarithmically in $Y_{\max }$. Quantitatively, at R=10 the window falls from roughly 7.6 months at L≈2 to 2.9 months at L≈77, a contraction by a factor of nearly three that is driven entirely by the redistribution of a fixed total burden across more lesions. Whether the window closes altogether depends on *R*, which is the least well constrained quantity in the framework: as reported in Sect. 7.6, closure occurs at R=10 only in the most fragmented corner of the parameter grid and does not occur at all for $R \ge 10^2$. We therefore do not claim that imaging is structurally incapable of detecting miliary disease. The defensible statement is that fragmentation compresses the window substantially, and that the compression is invisible to any model that tracks only aggregate mass.

This underscores the clinical necessity of longitudinal, serum-based physiological biomarkers over imaging. Biomarkers such as CA-125, when evaluated via the Risk of Ovarian Cancer Algorithm (ROCA), track the aggregate shedding mass *M*(*t*) rather than the spatial maximum $X_{\max }(t)$ (Bast et al. 2005). A patient with fragmented disease will register an elevated serum biomarker profile while the largest individual implant remains below the resolution limit, which is the content of prediction (P4) in Sect. 8.5 and highlights why multi-modal screening protocols out-perform ultrasound alone.

### Implications for Cytoreductive Surgical Strategies

The cornerstone of advanced ovarian cancer therapeutic management is maximal cytoreductive surgery, often categorized as primary debulking surgery (PDS) or interval debulking surgery (IDS) following neoadjuvant chemotherapy (NACT). Such therapy is administered with the objective of achieving complete resection to no gross residual disease (R0) (Vergote et al. 2010). The surgical resectability of a patient is highly dependent on the spatial topography of the metastases, a feature clinical tools like the Fagotti score attempt to quantify via laparoscopy (Fagotti et al. 2008).

Our model provides a quantitative basis for why the miliary phenotype correlates with high Fagotti scores and low rates of optimal cytoreduction. In the low-α bulky regime, the concentration of the metastatic mass into a small number of discrete sinks (L(∞) of order a few, as reported in Table 2) makes complete surgical excision anatomically feasible via standard procedures like omentectomy and pelvic peritonectomy.

In the high-α miliary regime, however, the fragmentation of the disease across many independent nodes (exceeding the twenty-implant threshold of Auer et al. (2015) that defines the phenotype) blanketing the small bowel serosa, mesenteric folds, and diaphragmatic surface makes complete surgical resection anatomically far more difficult, and frequently infeasible without causing life-threatening surgical morbidity (Torres et al. 2017). By mathematically defining how the growth-migration asymmetry permanently freezes this spatial landscape during early stages, our model suggests that pre-operative computational profiling of a patient’s metastatic topology (α estimation) could optimize the triage between immediate primary surgery and neoadjuvant chemotherapy.

### Therapeutic Disruption of Preferential Attachment Pathways

Finally, mapping this system to an infinite-color Polya urn highlights novel avenues for therapeutic intervention. Rather than focusing solely on cytotoxic agents that target cellular division rates ($\lambda _2$), our framework suggests that disrupting the preferential attachment signature could alter disease progression (Lengyel 2010).

The biochemical drivers of preferential attachment include cell adhesion molecules (CAMs) such as CD44 interacting with mesothelial hyaluronic acid, integrins ($\alpha _v\beta _3$ and $\alpha _5\beta _1$) binding to extracellular matrix components, and the mesothelin-CA125 binding axis (Davidson et al. 2012). Thermodynamically or pharmacologically blocking these attachment vectors would effectively suppress the migration capture term in our mean-field equations.

In terms of our model, neutralizing these adhesion pathways prevents arriving circulating cells from being captured by established tumor sinks, locking them instead in the peritoneal fluid suspension. Suspended cells are highly vulnerable to anoikis (suspension-induced apoptosis) and are more readily cleared by intraperitoneal chemotherapy regimes or localized immune surveillance. Thus, targeted anti-adhesion therapies could flatten the growth curves of dominant tumor sinks, slowing the approach to the radiological visibility threshold and extending the practical therapeutic window.

## Conclusion and Future Directions

This study extends the traditional multi-type branching process models of cancer by incorporating lesion-level heterogeneity via a Chinese Restaurant Process. We rigorously established that the growth rate discrepancies inherent to ovarian metastasis ($\lambda _2 > \lambda _1$) force the CRP to freeze, breaking standard Yule–Simon power laws and terminating de novo lesion formation. Using Polya urn martingales and fluid limits, we analytically defined the trajectories of individual lesions, and we validated each of these results quantitatively by direct stochastic simulation.

The analysis separates into what is a consequence of the modeling choice and what is not. That the model admits two regimes, one concentrated and one fragmented, is a consequence of the CRP parameterization: the seeding probability $\alpha /(M+\alpha )$ makes large α produce many lesions by construction. The freezing phenomenon is of a different character. It follows from the inequality $\lambda _2 > \lambda _1$, which is not a modeling choice but a consequence of the doubling times measured by Danesh et al., and it holds for every α. Holding α fixed and varying only the growth ratio, the expected lesion count rises from 7.9 at $\lambda _1/\lambda _2 = 0.3$ to 46.6 at $\lambda _1/\lambda _2 = 0.99$ and diverges as the ratio approaches unity, exactly as the exponent $p = 2 - \lambda _1/\lambda _2$ of the governing series approaches its critical value. The value 0.625 implied by the measured doubling times sits well inside the convergent regime. Freezing is thus a consequence of tumor kinetics, with α controlling only how many lesions form before the process halts.

The clinical implications are noteworthy: the 2.9-year window of opportunity calculated in prior literature implicitly assumes aggregate mass detectability. Our lesion-level model shows that, under its assumptions, fragmenting a fixed aggregate burden across more lesions contracts the screening window logarithmically in the dominant mass fraction, by a factor of roughly three across the range of lesion counts observed clinically. Whether the window closes entirely depends on the ratio *R* of lethal burden to imaging resolution, which our calibration suggests is larger than the value inherited from the aggregate-mass framework. We therefore report compression rather than categorical failure. These conclusions rest on a simplified description: the three-compartment exponential-growth structure and the assumption of a tissue-uniform α are idealizations, the CRP capture rule is an ansatz rather than a measured law (Sect. 3.5), and the parameters $\lambda _0, \lambda _1, \lambda _2, u_1, u_2$ carry substantial clinical uncertainty.

We identify three directions for further work. First, the predictions (P1)–(P4) of Sect. 8.5 are testable against operative and radiological records, and (P2) in particular requires no parameter estimation. Second, the model should be given genuine spatial structure: the present construction is exchangeable and therefore positionless, and replacing the uniform α by a site-dependent $\alpha (\textbf{x})$ over a realistic peritoneal geometry, distinguishing the omentum, the diaphragmatic surface and the small bowel serosa, and incorporating gravitational pooling of ascites, would convert a lesion-level model into a spatial one. Third, the capture rule itself invites empirical scrutiny: the CRP assumes capture linear in implant mass, and a sublinear rule would weaken the rich-get-richer effect and shift the phenotype boundary. Discriminating between these alternatives from serial imaging is an inference problem the framework poses but does not resolve.

Two technical questions also remain open. The non-degeneracy of the limiting mass fractions $Y_i^{(\infty )}$ is asserted in caveat (i) of Sect. 5.4 and supported numerically in Sect. 7.3, but not proved. The distribution of $Y_{\max }^{(\infty )}$, which our simulations show to be broad, is also not characterized analytically. Both appear tractable by adapting the theory of generalized Polya urns (Janson 2004) to the time-inhomogeneous setting.

## Data Availability

This manuscript has no associated experimental data. All simulation code, numerical output and figure-generating scripts supporting Sects. 7 and 8 are available from the author on request.
